# Image-based, whole-system hemodynamic modeling of mitral regurgitation and its impact on the right ventricular function

**DOI:** 10.3389/fcvm.2025.1673443

**Published:** 2026-01-30

**Authors:** Mia Bonini, Marc Hirschvogel, Maximilian Balmus, Yunus Ahmed, Hao Xu, Alistair Young, Paul C. Tang, David Nordsletten

**Affiliations:** 1 Department of Biomedical Engineering, University of Michigan, Ann Arbor, MI, United States; 2 Division 2.2 Process Simulation, Bundesanstalt für Materialforschung und -prüfung (BAM), Berlin, Germany; 3 School of Imaging Sciences and Biomedical Engineering, King’s College London, London, United Kingdom; 4 The Alan Turing Institute, London, United Kingdom; 5 Department of Cardiothoracic Surgery, Amsterdam University Medical Center, Amsterdam, Netherlands; 6 Department of Cardiovascular Surgery, Mayo Clinic, Rochester, MN, United States; 7 Department of Cardiac Surgery, University of Michigan Medical School, Ann Arbor, MI, United States

**Keywords:** computational fluid dynamics, hemodynamic modeling, lumped parameter modeling, mitral regurgitation, patient-specific modeling, right ventricle

## Abstract

**Introduction:**

Mitral regurgitation (MR) is a common valvular disease associated with complications such as pulmonary hypertension, atrial fibrillation, and heart failure. However, its full impact on the cardiovascular system, especially on right heart function, is not yet fully understood. Understanding this relationship is important because the right ventricle (RV) is critical for maintaining cardiovascular function. Dysfunction of the RV, which may be contributed by conditions like MR, is strongly associated with poor clinical outcomes. Despite its importance, comprehensively studying MR's effect on the RV has been challenging due to the complex, interdependent nature of cardiovascular dynamics, limited patient data, and the difficulty in synthesizing disparate information to clarify the left heart-right heart connection.

**Methods:**

The primary goal of this study is to investigate the effects of MR on cardiovascular hemodynamics and RV function by integrating 3D models of the left heart with a closed-loop 0D models of the entire cardiovascular system. We further conduct detailed analyses using patient-specific models to explore how various system modifications impact the RV, providing insights into the nuanced effects of MR on the right heart.

**Results and Discussion:**

This analysis provides several clinically relevant insights. First, progressive MR markedly increases RV afterload and predisposes the RV to dysfunction, even when intrinsic RV contractility is preserved or enhanced. Second, MR-specific severity indices and left-heart metrics alone fail to capture the true burden on the right heart; RV impairment can progress despite stable or only modestly changing MR descriptors. Finally, these findings highlight the need to incorporate direct assessment of RV structure and function into the evaluation of MR, as RV vulnerability plays a critical role in determining patient risk and guiding management decisions.

## 1 Introduction

Mitral regurgitation (MR) is the second most common valvular disease and affects more than 2% of the total population (1, 2). Secondary MR frequently develops as a consequence of heart failure–related ventricular remodeling, and if left untreated, MR can further worsen heart failure and ultimately result in death (3). Treatment of MR—through mitral repair, replacement or transcatheter edge-to-edge repair—has shown excellent outcomes (4); however, when patients should receive care to optimize outcomes remains challenging. Current guidelines for treatment focus predominantly on the valve and left heart (LH), aiming to capture whether the disease is primary or secondary as well as the cardiovascular impact of MR. However, the impact of MR on the right ventricle (RV) remains incompletely understood. In some cases, MR is correlated with a decreased RV ejection fraction, pulmonary hypertension, and heightened RV afterload (5–7). Furthermore, RV dysfunction (RVD) is frequent (61%) in MR patients and has been suggested as an independent predictor of survival in mitral valve (MV) surgical patients (5, 8, 9). However, discerning whether these outcomes primarily result from MR (and could be resolved with therapy) or indicate concurrence of MR and RV disease is unknown, and is further challenged by our incomplete understanding of the hemodynamic impacts of MR on the right heart.

Conventional clinical assessments, such as echocardiograms and cardiac catheterization, are commonly employed to establish correlations between cardiovascular data and MR outcomes (5, 10–12). While these methods provide valuable insights, computational fluid dynamics (CFD) modeling offers a complementary approach by enabling a mechanistic understanding of how MR affects RV function, capturing detailed flow dynamics and pressure changes that may not be directly measurable in clinical studies. Additionally, CFD allows for the isolation of MR’s impact on specific regions of the cardiovascular system, providing a clearer picture of localized hemodynamic changes that contribute to RV dysfunction. (13–15). Medical imaging provides patient-specific geometry and boundary motion that can be integrated into the 3D CFD model to enable the capture of complex cardiac deformations (13, 14, 16–19). The 3D CFD model can be coupled with reduced-order models (so-called 3D-0D modeling) to form a full circulatory model which is necessary for right and left heart analysis (14, 20). 3D-0D models allow for adaptive feedback to the 3D CFD model and provides a comprehensive look across the entire cardiovascular system.

This paper aims to quantitatively study the impact of mitral regurgitation MR on the RV using a combination of data, image analysis, and patient-specific hemodynamic modeling. To accomplish these objectives, we constructed a fully patient-specific 3D model of the left heart and aortic root using CT-derived boundary motion and coupled it to a closed-loop 0D circulation model. Clinical data were then assimilated to calibrate all 0D parameters, yielding a personalized whole-system representation that captures patient-specific anatomy, motion, and global hemodynamics. We applied this patient-specific 3D–0D framework to two MR cases and systematically varied EROA and RV contractility to isolate their hemodynamic effects. By evaluating a comprehensive set of RV-focused metrics, we found that worsening MR consistently compromises RV function, even when intrinsic RV contractility is enhanced. These results highlight the importance of examining RV responses directly, as traditional left-heart or MR-specific metrics do not fully capture the right-sided burden imposed by MR.

## 2 Materials and methods

### 2.1 Medical imaging and patient data

For this study, we collected data from two patients with mitral regurgitation. Patient A was a male with mild ventricular secondary MR, a heart rate of 53 bpm, and a height and weight of 193 cm and 121.5 kg, respectively. Patient B was a male with severe ventricular secondary MR, a heart rate of 120 bpm, a height of 165 cm, and a weight of 70.7 kg. Both patients had pulmonary hypertension and mild tricuspid regurgitation. For each patient, we collected data from their sphygmomanometer, cardiac catheterization, and echocardiogram exams as seen in Table 1. Dynamic computed tomography (4D CTA) images were also acquired to capture the motion of the left heart chambers. Both patients had CT images acquired on 64-detector CT scanners using helical acquisition mode (GE Medical System Discovery CT750 HD) with a spatial resolution of 0.4886 × 0.4885 × 0.625 mm in the sagittal, coronal, and axial directions, respectively. Images were acquired during intravenous injection of iopamidol and retrospective ECG-gating was used. The dynamic CT images for Patients A and B consisted of 10 and 20 time frames, respectively, covering the full cardiac cycle. Institutional review board approval was obtained (protocol No. HUM00196629, April 2021), and no informed consent was required.

**Table 1:** Exam results for patients A and B.

Exam	Data	Patient A data	Patient B data
Sphygmomanometer	Sys. sBP (mmHg)	117.0	128.0
	Sys. dBP (mmHg)	76.0	87.5
Cardiac catheterization	Pul. sBP (mmHg)	39.0	67.0
	Pul. dBP (mmHg)	19.0	43.0
	RV sBP (mmHg)	37.0	60.0
	RV dBP (mmHg)	10.0	17.0
	LV EDP (mmHg)	19.0	40.0
	RV EDP (mmHg)	14.0	27.0
	Mean RA Pres. (mmHg)	10.0	17.0
	Max LA Pres. (mmHg)	21.0	53.0
	Max RA Pres. (mmHg)	12.9	21.0
Echocardiogram	MRF	0.22	0.5
Dynamic CT imaging	LV SV (mL)	88.8	65.0
	LVESV (mL)	132.8	340.0
	LVEDV (mL)	221.6	405.0
	LA SV (mL)	29.8	31.8
	LA Min. Vol. (mL)	110.7	279.9
	LA Max. Vol. (mL)	140.5	311.7
	RVEDV (mL)	261.3	285.1

Syst., systemic; sBP, systolic blood pressure; dBP, diastolic blood pressure; Pulm., pulmonary; LVEDP, left ventricle end diastolic pressure; RVEDP, right ventricle end diastolic pressure; Pres., pressure; MRF, mitral regurgitant fraction; SV, stroke volume; Vol., volume.

### 2.2 Anatomic reconstruction and blood flow segmentation

**Figure 1:**
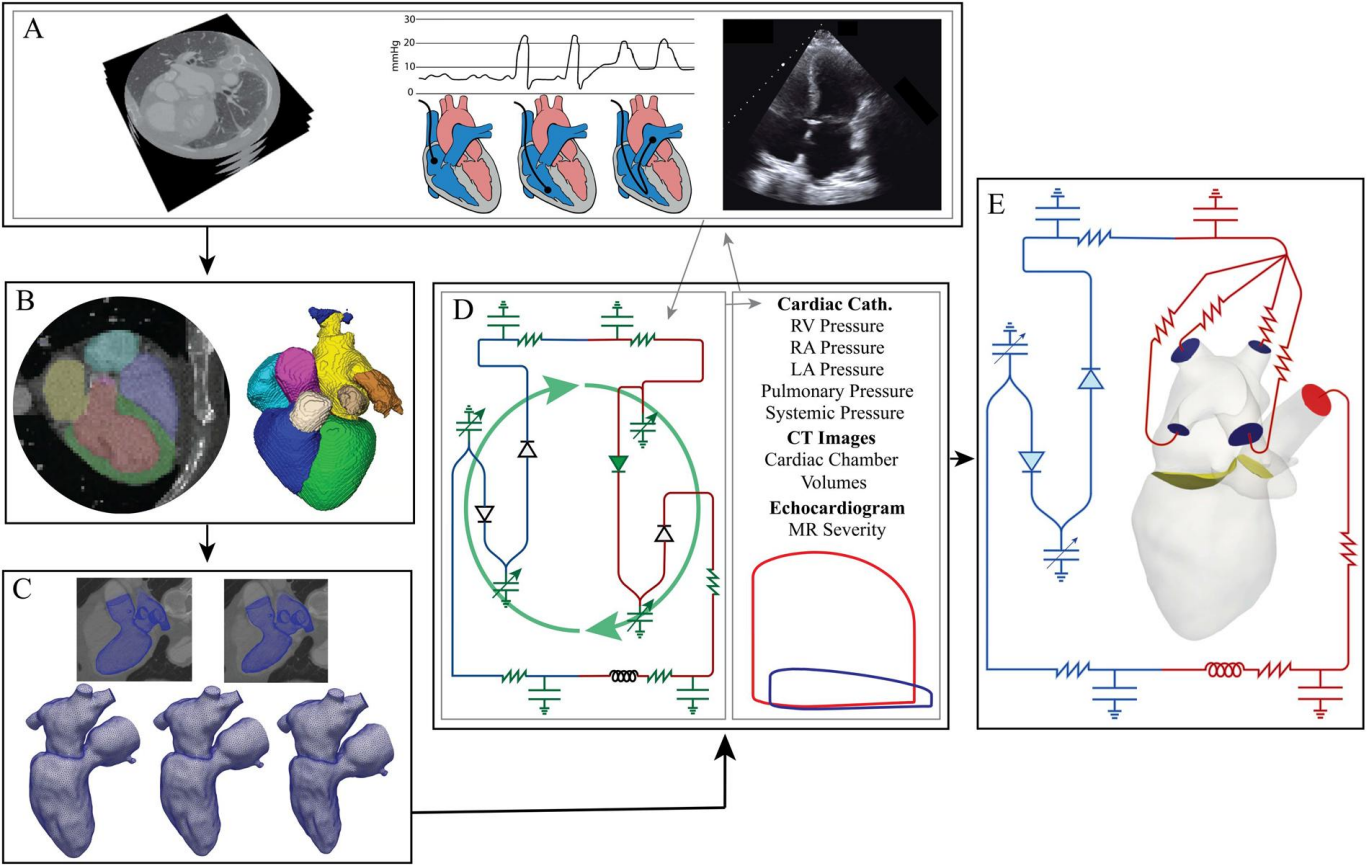
Modeling pipeline: **(A)** data collection (left to right: dynamic CT images with contrast, cardiac catheterization, echocardiograms). **(B)** Neural network segmentation and then manual editing in 3D Slicer. **(C)** Tetrahedral volume meshing of the LV, LA, and aortic root and boundary deformation calculation performed in Simmodeler (22). **(D)** 0D parameter optimization using patient data. Gray lines indicate the data used to determine the optimal 0D parameters. The green, curved arrows signify that the green electrical circuit symbols are updated during the optimization process. **(E)** 3D-0D CFD modeling.

Patient-specific anatomies were reconstructed from axial CT images (Figure 1A). A 3D U-Net-based neural network was used to segment the blood volume of the LV, LA, pulmonary veins, and aortic root (Figure 1B) (21). Manual segmentation edits were performed in 3D Slicer to extend the aortic root, add coronary arteries, and extract the aortic and mitral valve annuli. Segmentations were saved as discrete data and imported into SimModeler to smooth the model and create tetrahedral volume meshes and 3D boundary layers (Figure 1C) (22). Tetrahedral volume meshes were generated with 1.05 M elements for patient A and 1.03 M elements for patient B.

To create a mesh for each time point in the CT data, the computational domain was imported into Eidolon for motion-tracking from the 4D CTA (23). This procedure used IRTK’s built-in routines, based on non-rigid registration, generating deformation fields for each time phase in the CT images (24, 25). The deformation was applied to the initial mesh and resulted in deformed meshes for each time phase in the CT images (Figure 1C). Temporal interpolation was performed on the mesh series, and the resulting displacements were used to calculate domain wall velocity ($v_{D}$). Next, the boundary deformations were propagated through the 3D domain using a linear elastic problem that provided a field, $\hat{v}(t)$, describing the domain velocity as well as the state of the domain Ω(t) at each time point t∈[0,T] (26).

### 2.3 Data assimilation

**Figure 2:**
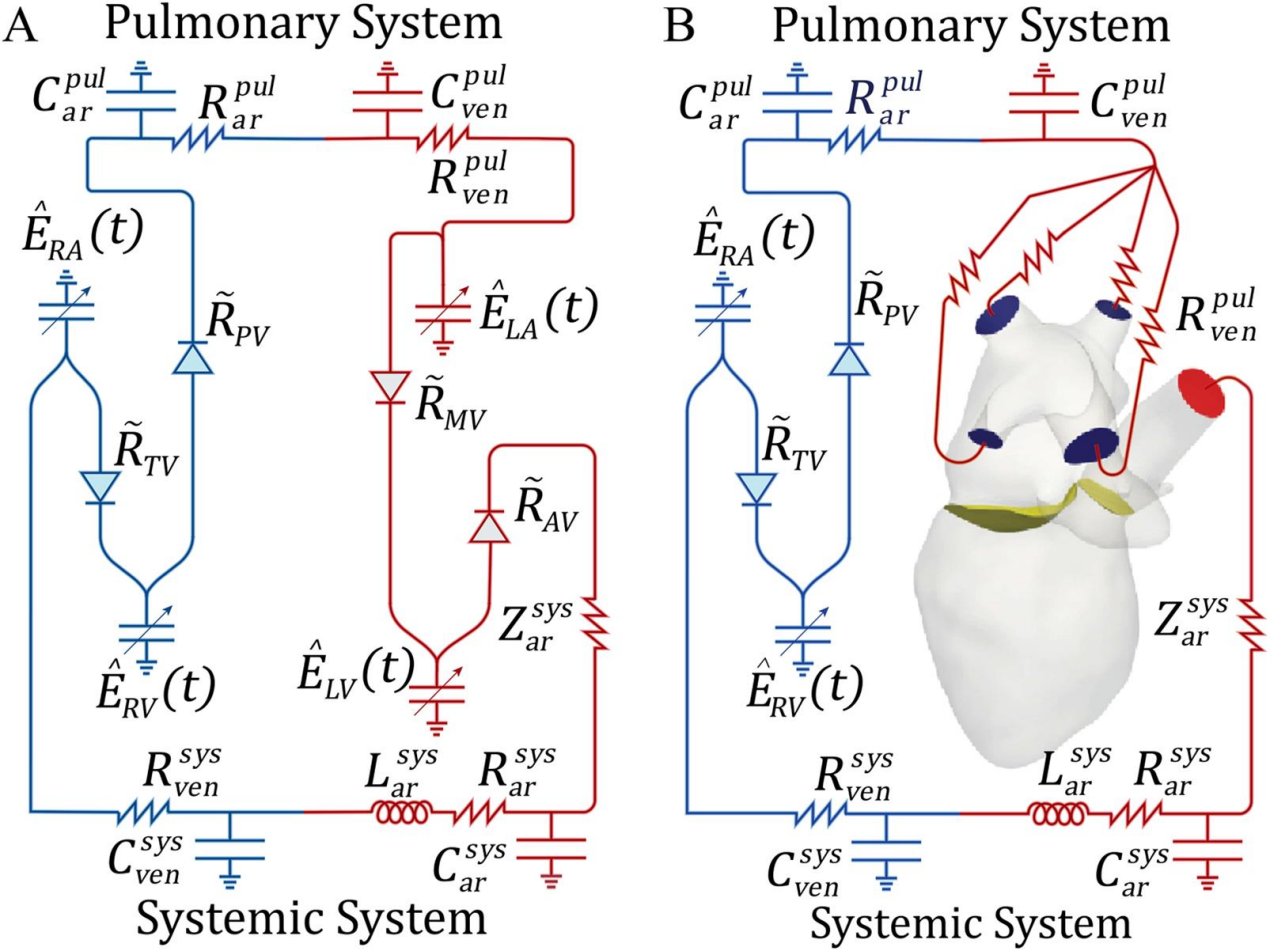
Schematic representations of the 0D Windkessel and 3D-0D models: **(A)** 0D model used for parameter optimization, **(B)** 3D-0D closed loop model in which Neumann BCs are applied to the aortic root outflow face ($\Gamma_{t}^{AO}$, red) and pulmonary veins ($\Gamma_{t}^{PV}$, blue) and coupled to the 0D model with a Lagrange multiplier. The flow is controlled by the mitral ($\Gamma_{t}^{MV}$) and aortic valves ($\Gamma_{t}^{AV}$) shown in yellow. *R* or *Z*, resistance; *C*, capacitance; *L*, inductance; $\hat{E}(t)$, time varying elastance; pul, pulmonary; sys, systemic; ven, veins; ar, arteries; $\tilde{R}$, pressure dependent valve resistance; RV, right ventricle; RA, right atrium; LV, left ventricle; LA, left atrium; TV, tricuspid valve; PV, pulmonary valve; MV, mitral valve; AV, aortic valve.

A zero-dimensional model was employed to evaluate the impact of MR on the RV and the remaining cardiovascular system. The 0D model consisted of the systemic and pulmonary circulations modeled using resistance-inductance-capacitance (RLC) circuits. The lumped-parameter model captured the key physiological conditions of the cardiovascular system. The resistance modeled the viscosity effects, the capacitance modeled the compliance of the vessels, and the inductance modeled the inertial effects. The cardiac chambers were modeled using a time-varying linear elastic model. More information about this model, including equations, can be found in the Supplementary Material. We characterized and fit parameters in the 0D model, θ (comprised of the 18 variables of the 0D model defining the *R, C, E* and *Z* in Figure 2A), using the patient data shown in Table 3. The MR severity classification was known for Patients A and B from their echocardiogram results, which we used to determine the mitral regurgitant fraction (MRF), with MR severity mild (MRF 0.22) and severe (MRF 0.50).

To solve for all 0D model parameters, we employed a combination of a gradient descent algorithm and a Levenberg-Marquardt optimization algorithm to minimize the following least-squares objective function (Equation 1),

$$J(\theta )=\sum_{i=1}^{N}\frac{1}{2}{\left(\frac{{\hat{y}}_{i}-y_{i}}{y_{i}}\right)}^{2},$$ (1)

where $\hat{y_{i}}$ are the 0D solution features that we calibrated against, y was the patient data, and i looped through the N patient measurements (see the first column in Table 1). Each model ran for 15 iterations, with convergence defined as J<0.1. The final 0D Windkessel parameters are provided in the Supplementary Material.

**Figure 3:**
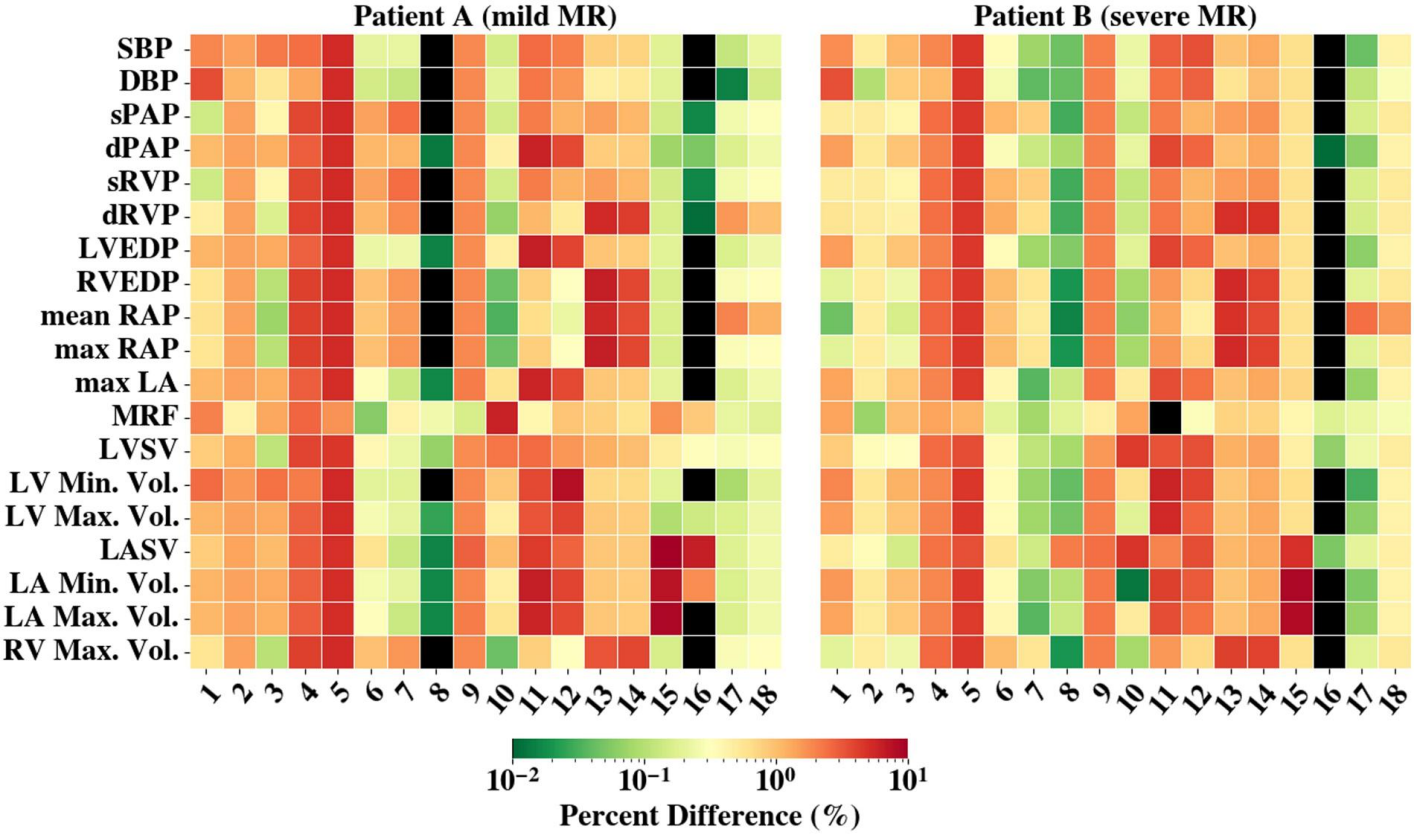
The averaged percent difference between the final fit model results and perturbed 0D model (±10%) results are shown. The colorbar is scaled logarithmically. Black shading represents values with less than 0.01% difference. The *y*-axis denotes the compared results and *x*-axis are the perturbed parameters (1-$R_{\mathrm{ar}}^{sys}$, 2-$C_{\mathrm{ar}}^{sys}$, 3-$Z_{\mathrm{ar}}^{sys}$, 4-$R_{\mathrm{ven}}^{sys}$, 5-$C_{\mathrm{ven}}^{sys}$, 6-$R_{\mathrm{ar}}^{\;pul}$, 7-$C_{\mathrm{ar}}^{\;pul}$, 8-$R_{\mathrm{ven}}^{\;pul}$, 9-$C_{\mathrm{ven}}^{\;pul}$, 10-$MV_{\mathrm{ROA}}$, 11-$E_{\min}^{LV}$, 12-$E_{\max}^{LV}$, 13-$E_{\min}^{RV}$, 14-$E_{\max}^{RV}$, 15-$E_{\min}^{LA}$, 16-$E_{\max}^{LA}$, 17-$E_{\min}^{RA}$, 18-$E_{\max}^{RA}$) Abbreviations can be found in Figure 2 and Table 3 captions. RAO, regurgitant orifice area.

We assessed the sensitivity of our 0D model by perturbing each parameter by ±10% and measuring the percent change in outputs. The averaged results, shown in Figure 3, indicated that all parameters influenced model behavior, yet none exhibited extreme sensitivity (all changes <15%). This suggested the model was well-conditioned, with no single parameter dominating, and that parameter fitting effectively captured key physiological dynamics.

To integrate the 3D model with the 0D model representation and expedite convergence to a steady state, we re-ran the 0D model using the chamber volumes computed from tracking of the 4D CTA. Specifically, we replaced the 0D elastance models of the LA and LV with the time-varying, patient-specific volumes for each chamber, respectively. We then ran the 0D Windkessel model until it achieved periodic steady state (70 cardiac cycles with <1% change in pressure and flow between cycles). The 0D parameters obtained from the optimization process were directly used to define the 0D model parameters in the 3D-0D coupled simulation (Figure 2B).

### 2.4 Hemodynamic flow problem

To predict flow for different patient cases, we utilized the finite-element-based flow solver, C**Heart** (27). An example of the modeled domain and boundaries is shown in Figure 1. We determined pressure (p) and blood velocity (v) by solving the Arbitrary Lagrangian–Eulerian Navier-Stokes equations (Equations 2–9) within the domain Ω(t) (26, 28). These equations were: ?>

$$\rho \frac{\partial v}{\partial t}+\rho \left[(v-\hat{v})\cdot \nabla \right]v-\nabla \cdot \sigma =0,\;\text{on}\;\Omega ,$$ (2)

$$\Phi (v-\hat{v})=0,\;\text{on}\;\Gamma_{\mathrm{AV}},$$ (3)

∇⋅v=0,onΩ, (4)

$$v=\hat{v},\;\text{on}\;\Gamma^{D},$$ (5)

$$\sigma \cdot n+\lambda_{j}n=0,\;\text{on}\;\Gamma_{0D,j}(t),\;j\in \{1,\ldots ,N^{0D}\},$$ (6)

$$M\frac{\partial y}{\partial t}-f(t,y)=0,$$ (7)

$$\int_{\Gamma_{0D}}(v-\hat{v})\cdot n\;dA-y\cdot {\hat{e}}_{Q,j}=0,\;j\in \{1,\ldots ,N^{0D}\},$$ (8)

$$y\cdot {\hat{e}}_{\;p,j}-\lambda_{j}=0,\;j\in \{1,\ldots ,N^{0D}\},$$ (9)

where ρ was the fluid density, $\hat{v}$ was the arbitrary domain velocity, $\sigma =\mu_{f}(\nabla v+\nabla v^{T})-pI$ was the Cauchy stress tensor, $\Gamma =\Gamma^{N}\cup \Gamma^{D}$ was the boundary of Ω, and $\mu_{f}$ was the bulk viscosity. We prescribed a no-slip boundary condition (BC) on the wall of the LA, LV, and aortic root sub-domains. A Dirichlet condition was used to define the motion of the LV, LA, and aortic wall $\left(\Gamma^{D}\right)$.

Equation 6, enforces the coupling of the 3D-0D sub-domains with Lagrange multiplier constraints (λ) used to define the traction over each inflow/outflow boundary (shown in blue and red, Figure 2B). Here, $N^{0D}=5$ is the number of coupling faces between the 3D and the 0D model ($\Gamma^{PV}\cup \Gamma^{AO}$). The 0D lumped parameter model is denoted by Equation 7. Here, y are the state variables, M is a matrix that contains rate dependent terms, and f(y) collects the other contributions. Equations 8, 9 ensure that the pressure and flow at the 3D model faces ($\Gamma^{PV}\cup \Gamma^{AO}$) and the pressure and flow in the respective 0D model compartment, are equivalent. ${\hat{e}}_{Q,j}$ and ${\hat{e}}_{\;p,j}$ denote unit vectors that isolate 0D model variables for flow, Q, and pressure, p, at indicated boundaries j.

We modeled the mitral (MV) and aortic (AV) valves in open and closed configurations. The timing of MV opening and closure was tied to the cardiac phase, determined from changes in LV volume derived from the boundary-driven mesh. During systole, a Dirichlet condition was applied over most of the MV plane to restrict flow, while mitral regurgitation was represented by permitting flow through a prescribed region such that the effective regurgitant orifice area (EROA) matched the patient’s clinical MR severity (2). Flow through this region was fully resolved by the Navier–Stokes equations. During diastole, no boundary condition was imposed on the MV, and transvalvular flow arose from the Navier–Stokes solution. MV opening and closure were assumed to occur instantaneously (29).

Due to mitral regurgitation, defining AV opening and closing times based on LV volume change led to nonphysical inflow. Instead, we incorporated a penalty function (Φ) into the momentum equation at the aortic valve plane, expressed as $\Phi (v-\hat{v})$ (30). This function enforced resistance-based flow control by assigning a temporally varying resistance value, Φ, which varied between 0 (to allow flow) and $10^{6}$ (to restrict flow): $\Phi (t)=10^{6}(1-g(t))$. The dynamic behavior of the valves was determined by the solution (g) of the ordinary differential equation (ODE) given in Equation 10. A fully open valve corresponded to g=1, while a fully closed valve corresponded to g=0. The governing ODE was defined as:

$$\frac{dg}{dt}=\frac{C_{1}(\alpha )}{2}[1+\alpha (1-2g)],\;\alpha =\tanh(C_{2}\cdot \Delta P+C_{3}\cdot Q_{\mathrm{AV}}),$$ (10)

Here, the rate of change of g was determined by α∈(−1,1), which depended on the pressure gradient (ΔP), transvalvular flow (Q), and its rate of change. A large positive pressure gradient or forward flow shifted α→1, leading to rapid valve opening as g approached 1. Conversely, a significant negative pressure or backflow shifted α→−1, driving g toward 0 (closed state). We computed the pressure drop $\Delta P=P_{\mathrm{lv}}-P_{\mathrm{aorta}}$ using spatially averaged upstream and downstream pressures. We determined the transvalvular flow $Q_{\mathrm{AV}}$ by integrating the flux across the aortic valve plane. The parameter $C_{1}=883$ was chosen to regulate the time scale of valve movement, ensuring full opening or closure occurred within 1% of the cardiac cycle. We selected empirical values of $C_{2}=0.005$ $Pa^{-1}$ and $C_{3}=0.25\;s\cdot {\mathrm{mm}}^{-3}$ to maintain stable valve dynamics.

We coupled the pulmonary veins ($\Gamma_{t}^{PV}$) and aortic root outflow ($\Gamma_{t}^{AO}$) boundaries to a reduced-order zero-dimensional (0D) vascular system model to create a closed-loop multiscale model (20). We solved the final 3D-0D CFD model using a strongly coupled monolithic framework, where all governing equations were solved simultaneously to maintain consistency and accuracy across the coupled domain. At each Newton iteration, we updated variables to ensure convergence. We imposed a convergence tolerance of $10^{-6}$ to ensure numerical accuracy. To enhance numerical stability, we applied a stabilization scheme proposed by Hoffmann et al. (31), utilizing $P^{1}-P^{1}$ elements for fluid velocity and pressure. We initialized the model with zero velocity and zero pressure in the 3D domain, while the 0D model was initialized using the steady-state solution of a standalone 0D simulation described in Section 2.3 (Figure 2A). We ran simulations for three full cardiac cycles, ensuring a steady-state condition where changes in the 0D pressures and flows and mitral valve and aortic valve flows were below 5%.

### 2.5 Simulation framework for MR and RV interaction

These methods established models that replicated the behavior observed from the patient chart. From there, we began to examine the interplay between MR and RV function. To do so, we used the patient-specific case of mild MR as a baseline model and perturbed it to understand the impact of changes in the cardiovascular system. All perturbations were first run with the 0D model to reach steady state. The final pressures and volumetric fluxes were then used to initialize the 3D-0D model. In this work, we studied the effect of the following:

1. **Mitral regurgitant severity:** To study the effect of varying MR severity on the RV, we modeled an EROA of 0.18 ${\mathrm{cm}}^{2}$ (baseline), 0.30 ${\mathrm{cm}}^{2}$, and 0.60 ${\mathrm{cm}}^{2}$. These values fell within the clinical definitions of mild, moderate, and severe MR, respectively. Furthermore, evidence showed that pulmonary vascular resistance (PVR) was positively correlated with MR severity (32, 33). Therefore, models with an EROA of 0.18 ${\mathrm{cm}}^{2}$, 0.30 ${\mathrm{cm}}^{2}$, and 0.60 ${\mathrm{cm}}^{2}$ were prescribed a total PVR of 1.25 Wood Units (WU), 3.75 WU, and 6.25 WU, respectively. This was achieved by adjusting the pulmonary resistance parameters ($R_{\mathrm{ar}}^{\mathrm{pul}},R_{\mathrm{ven}}^{\mathrm{pul}}$) in the 0D model (Figure 2).
2. **RV contractility:** To account for variations in RV functionality, we adjusted the RV maximum elastance, a parameter that influenced the contractility of the RV (34). We increased the baseline model’s RV maximum elastance ($E_{r,\max}^{v}$) from 0.18 mmHg/mL to 0.7 mmHg/mL. More information on the 0D elastance model and how elastance defined chamber function is discussed in the Supplementary Material.

By systematically making these changes to the baseline model, we gained a mechanistic understanding of the interaction between MR, RV function, and their impact on the broader cardiovascular system. Table 2 defines the variables that were calculated to quantify the impact of these changes.

**Table 2:** Hemodynamic variables used to quantify the severity of MR and risks to the RV.

Variable	Equation	Description
Pulmonary artery pressure (PAP)	–	A mPAP >= 25 mmHg signifies PHT; sPAP > 50 mmHg is severe PHT and is a determinant of RV dysfunction (6).
LV end diastolic pressure (LVEDP)	–	normal: LVEDP < 15 mmHg; moderately abnormal: 15–30 mmHg; markedly elevated (LVEDP > 30 mmHg) (35).
Central venous pressure (CVP)	–	normal: 1–6 mmHg; elevated (>15 mmHg) indicates myocardial contractile dysfunction (36).
Passive cardiac index (PasCI)	$\frac{\mathrm{RAP}\cdot \mathrm{CO}}{\mathrm{mPAP}\cdot \mathrm{BSA}}$	High PasCI (>0.5) is correlated with reduced survival and increased risk of RV failure after surgery in LVAD patients (9)
RV ejection fraction (RVEF)	$\frac{\mathrm{RVSV}}{\mathrm{RVEDV}}$	RVEF < 45% defined as RV dysfunction (5)
Mitral regurgitant fraction (MRF)	${\frac{\mathrm{MVRV}}{\mathrm{MVSV}}}_{f}$	Mild: MRF < 30%; moderate: MRF = 30%–49%; severe: MRF > 50%
Transpulmonary pressure gradient (TPG)	mPAP−mPCWP	TPG > 12 mmHg signifies reactive PHT which can cause compensatory RV myocardial hypertrophy and further dysfunction (8)
Pulmonary artery pulsitility index (PAPi)	$\frac{\mathrm{sPAP}\;-\;\mathrm{dPAP}}{\mathrm{CVP}}$	Reflects RV preload and afterload; PAPi < 1.85 suggests risk of RVF (9, 37)
Right ventricle stroke work index (RVSWI)	$\frac{\mathrm{CO}(\mathrm{mPAP}-\mathrm{RAP})\cdot 0.0136}{\mathrm{BSA}}$	RVSWI outside of the normal range [5–10 ${\text{g/m}}^{2}$/beat (38) or 8–12 ${\text{g/m}}^{2}$/beat (39)] is associated with poor outcomes.

sPAP, systolic pulmonary artery pressure; dPAP, diastolic pulmonary artery pressure; PHT, pulmonary hypertension; LVEDP, LV end diastolic pressure; RAP, right atrium pressure; MVRV, mitral valve regurgitant volume; ${\mathrm{MVSV}}_{f}$, mitral valve forward stroke volume; mPAP, mean pulmonary artery pressure; mPCWP, mean pulmonary artery capillary wedge pressure; BSA, body surface area; CO, cardiac output.

## 3 Results

### 3.1 Patient-specific results

**Figure 4:**
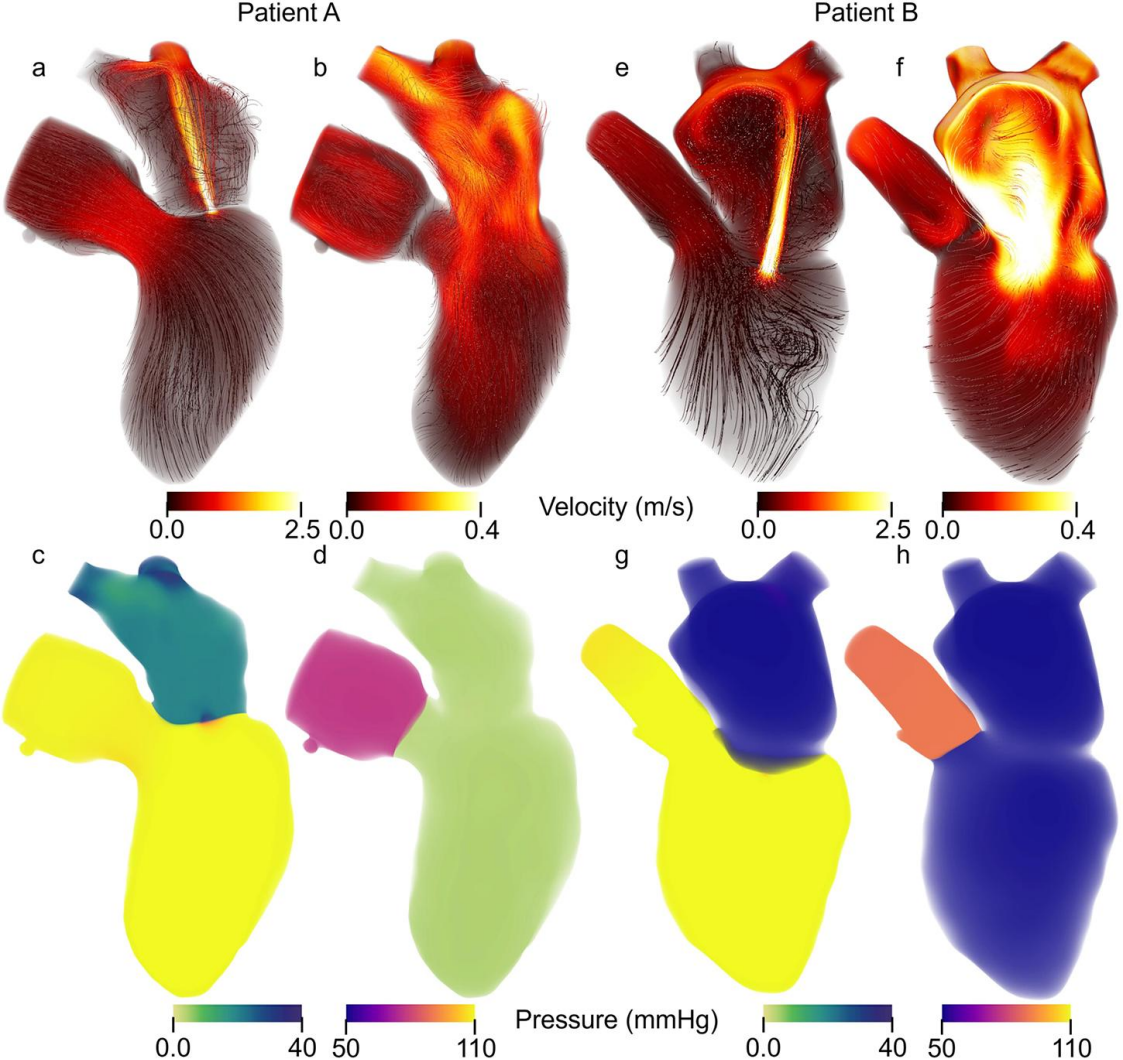
Patient specific Velocity and pressure fields during peak systole and diastole. Streamlines colored according to velocity magnitude for Patient A during **(a)** peak systole and **(b)** peak diastole as well as Patient B at **(e)** peak systole and **(f)** peak diastole. Note that the velocity range is the same for peak systole between patients A and B but different for peak diastole. Volume rendering of the pressure for Patient A at **(c)** peak systole and **(d)** peak diastole and for Patient B and **(g)** peak systole and **(h)** peak diastole.

In this work, we modeled 2 patient-specific cases of mitral regurgitation: mild (Patient A) and severe (Patient B). The flow and pressure fields from the 3D-0D results for Patients A and B at peak systole and diastole are shown in Figure 4. Periodic steady-state was determined when there was less than a 5% difference between cardiac cycle results. The results of the 0D optimization problem, explained in Section 2.3, are shown in Table 3. The shading in Table 3 denotes the percentage error relative to the patient data.

**Table 3:** Patient specific results for model validation compared to Patient A data and Patient B data. The results of the 0D and 3D-0D models are shaded to indicate percentage error. In the case of Patient A, poor contrast in the right heart did not allow for measurement of the right ventricle blood volume.

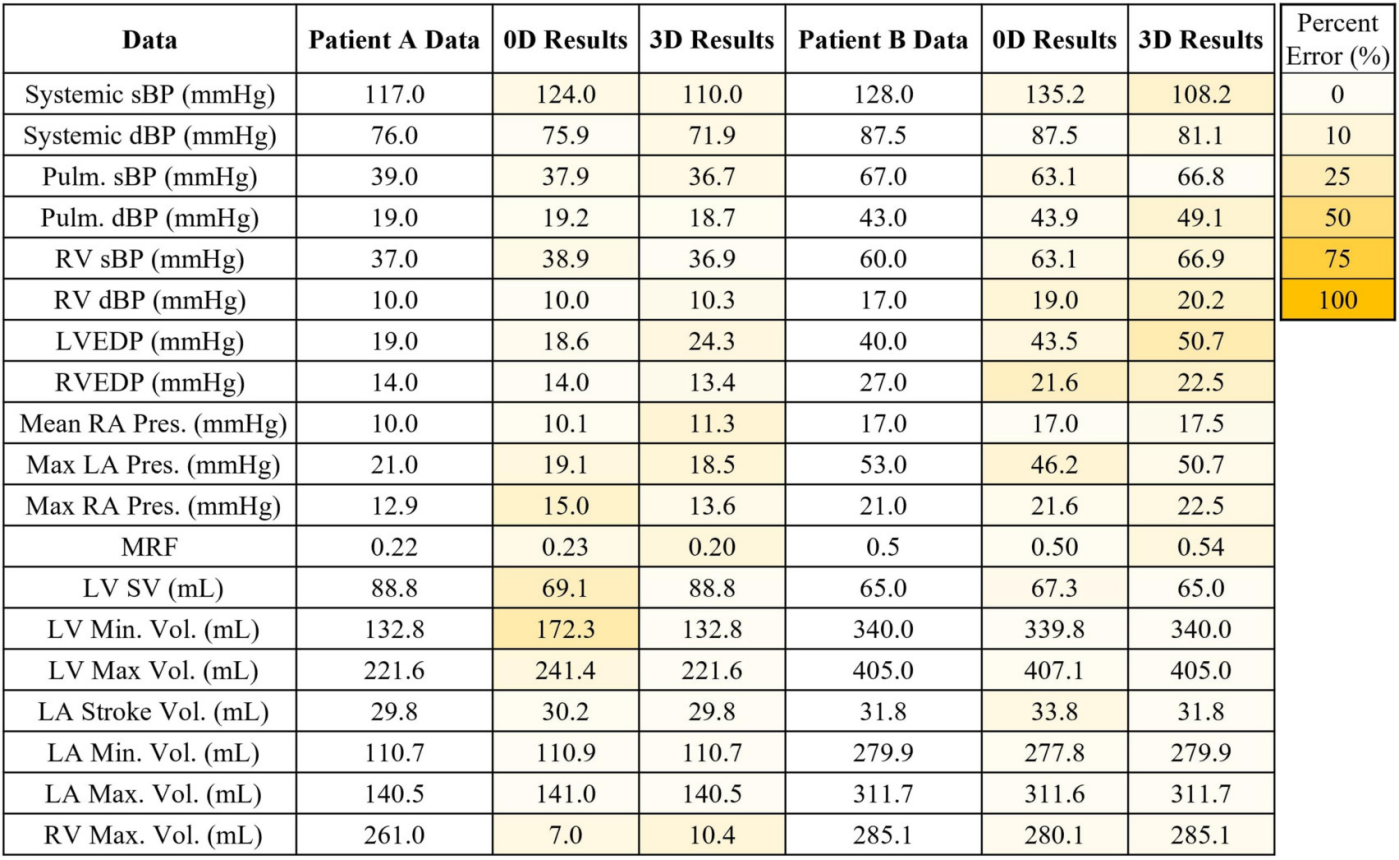

**Figure 5:**
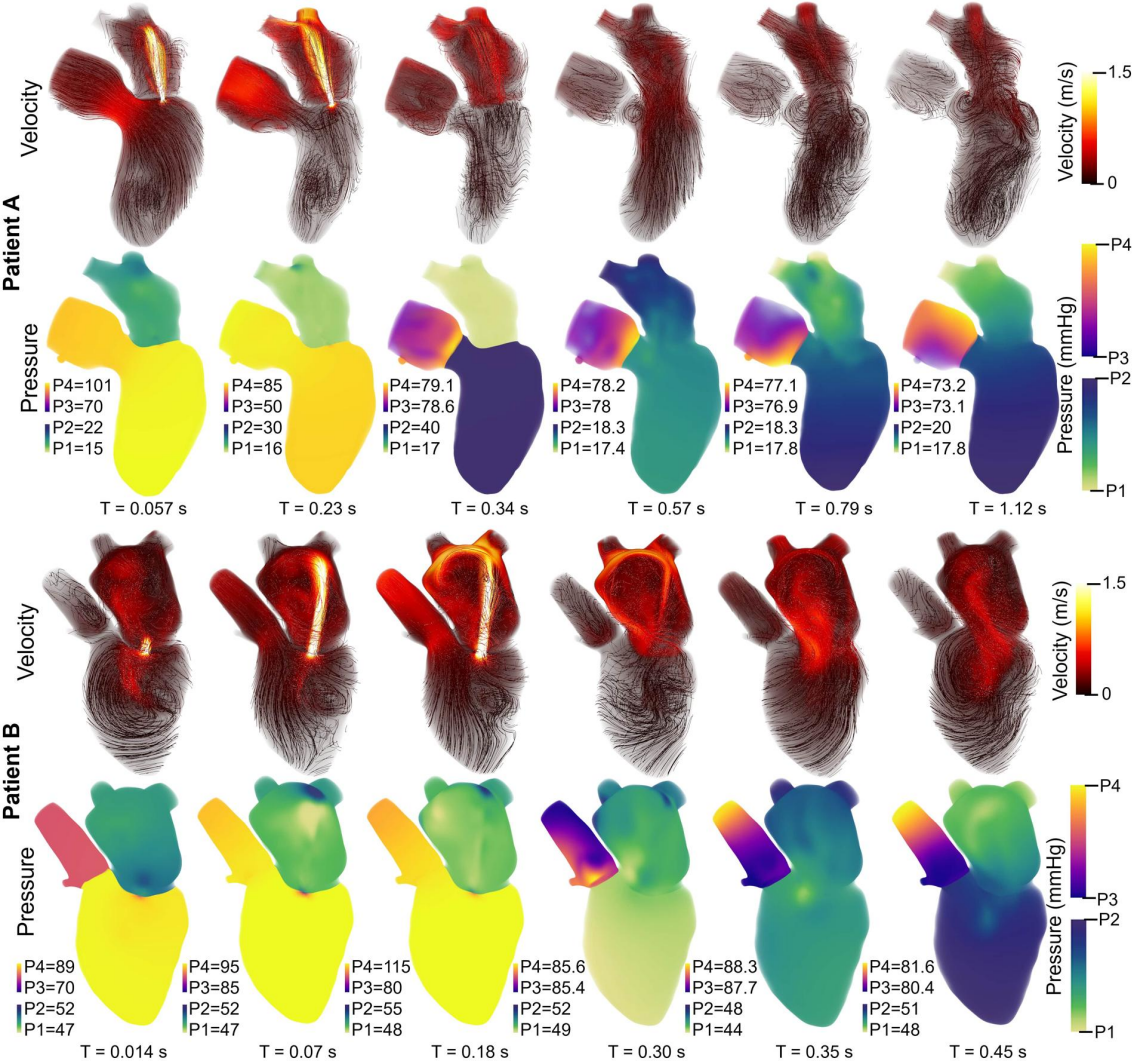
Velocity streamlines (colored by magnitude) and pressure fields with volume rendering for Patient A (upper panel) and Patient B (lower panel) over the cardiac cycle. The pressure colorbar ranges vary and are indicated to the left of each sub-image.

Patient A model results agreed well with the patient data. The average percent error between the computational results and patient data is 6.2% and 7.8% for the 0D and 3D-0D results, respectively. The flow and pressure over the cardiac cycle is displayed for this patient in Figure 5. During systole a regurgitant jet was observed, reaching up into the LA to the pulmonary veins and disrupting the flow. At peak systole, the highest pressures are seen in the LV and aortic root of 112 mmHg, which decreases as end systole approaches. During diastole, we observed a ventricular vortex formation which is expected to occur during filling (14, 16). Results also show a gradual increase in pressure and volume in the LV throughout diastole. Patient A had a regurgitant volume of 17.6 mL and a peak regurgitant jet velocity of 4.12 m/s (Figure 4a). During diastole, Patient A had an E-wave velocity of 0.32 m/s and an A-wave velocity of 0.29 m/s (Figure 4b). In Patient A there was flow reversal, during systole, in two pulmonary veins with a total of 17.6 mL. The maximum pressure in the LV for Patient A was 112 mmHg and the LVEDP was 24.3 mmHg (Figures 4c, d).

Patient B, with severe MR, shows results that also agree well with the data. The average percent error between the computational results and patient data was 4.7% and 10.3% for the 0D and 3D-0D results respectively. Patient B had a large regurgitant volume of 34.4 mL and a peak regurgitant jet velocities of 4.0 m/s respectively (Figure 4e). During diastole, Patient B’s E-wave velocity was 1.0 m/s and had no recognizable A-wave velocity (Figure 4f). This aligns closely with the patient data which reported a regurgitant velocity of 3.9 m/s, an E-wave velocity of 1.55 m/s, and no A-wave velocity. The E-wave velocity in the patient data was likely higher than in our model because our model allowed flow through the entire mitral valve plane, whereas in reality, the mitral valve leaflets create a smaller effective opening area during diastole, which would lead to higher flows. However, our model did capture an E-wave greater than 0.9–1.2 m/s, which is strongly correlated with severe MR (12, 40). Patient B had flow reversal in all 4 of the pulmonary veins during systole with a total pulmonary vein flow reversal of 7.5 mL. The maximum pressure in the LV for Patient B was 116 mmHg and the LVEDP was 51 mmHg (Figures 4g, h)

### 3.2 Mitral regurgitation severity

**Figure 6:**
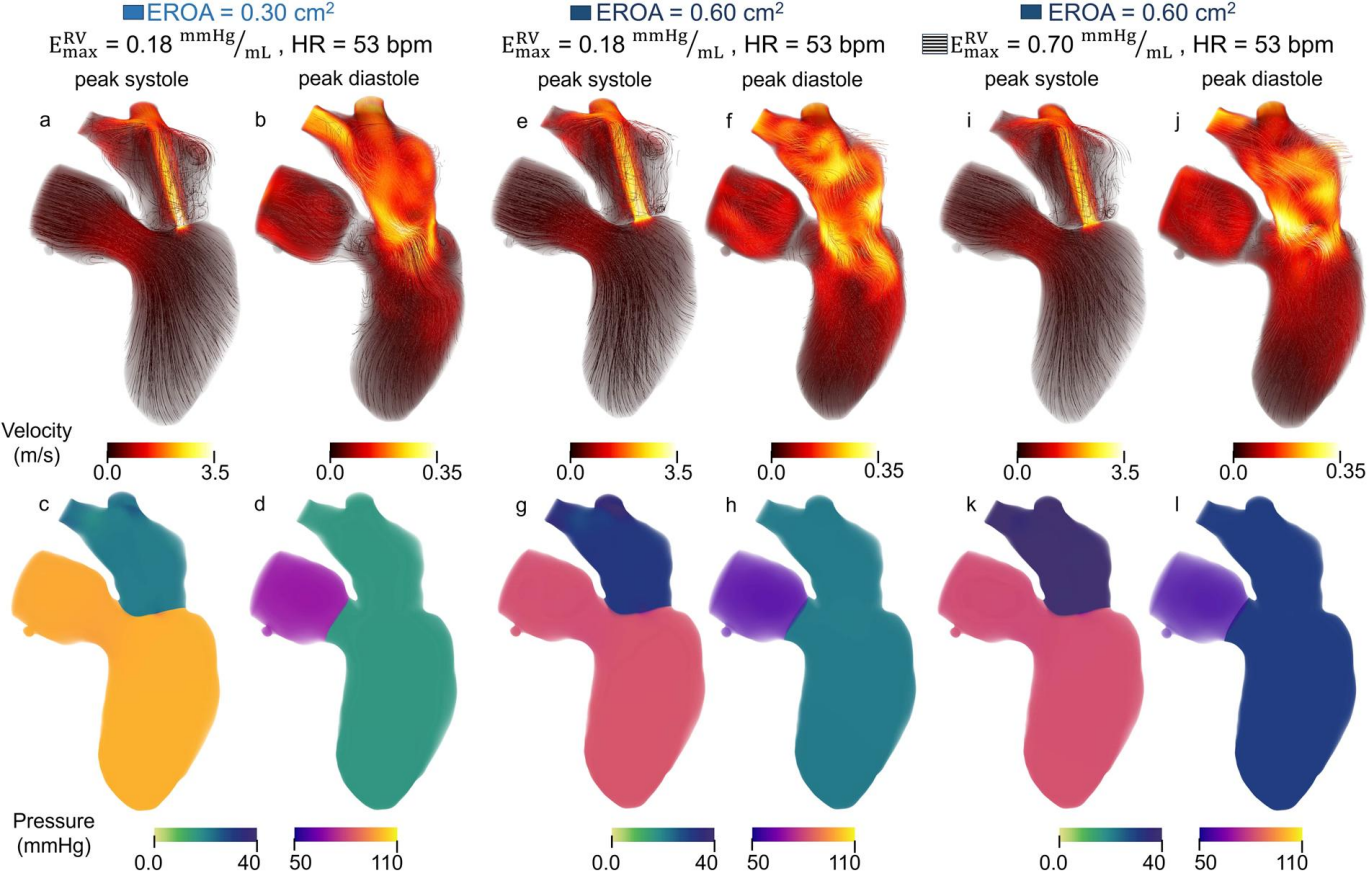
Velocity and pressure during peak systole and mid diastole for models with **(a–d)** moderate EROA and maximum RV elastance of 0.18 mmHg/mL, **(e–h)** moderate EROA and maximum RV elastance of 0.70 mmHg/mL, **(i–l)** severe EROA and maximum RV elastance of 0.70 mmHg/mL. Streamlines are colored according to velocity magnitude and pressure is visualized with volume rendering.

The impact of EROA on left-heart flow patterns is shown in Figures 6a, c. Larger EROAs produced wider regurgitant jets and increased backflow into the left atrium. Increasing the EROA from mild to moderate resulted in a 74% increase in mitral regurgitant volume and a 2.6% decrease in peak regurgitant jet velocity, while increasing EROA from mild to severe resulted in a 144% increase in regurgitant volume accompanied by a 13.4% decrease in peak regurgitant jet velocity. Higher E-wave velocities were also observed with larger EROAs. From moderate to severe EROA, the E-wave velocity increased by 27.8% (Figures 6b vs. d). A larger EROA led to lower cardiac output and lower systolic pressure in the aortic root (Figures 6g vs. i), as well as slightly higher filling pressures in the left atrium and ventricle (Figures 6h vs. j). We also evaluated the impact of an increased heart rate to maintain cardiac output. This analysis can be found in the Supplementary Material.

**Figure 7:**
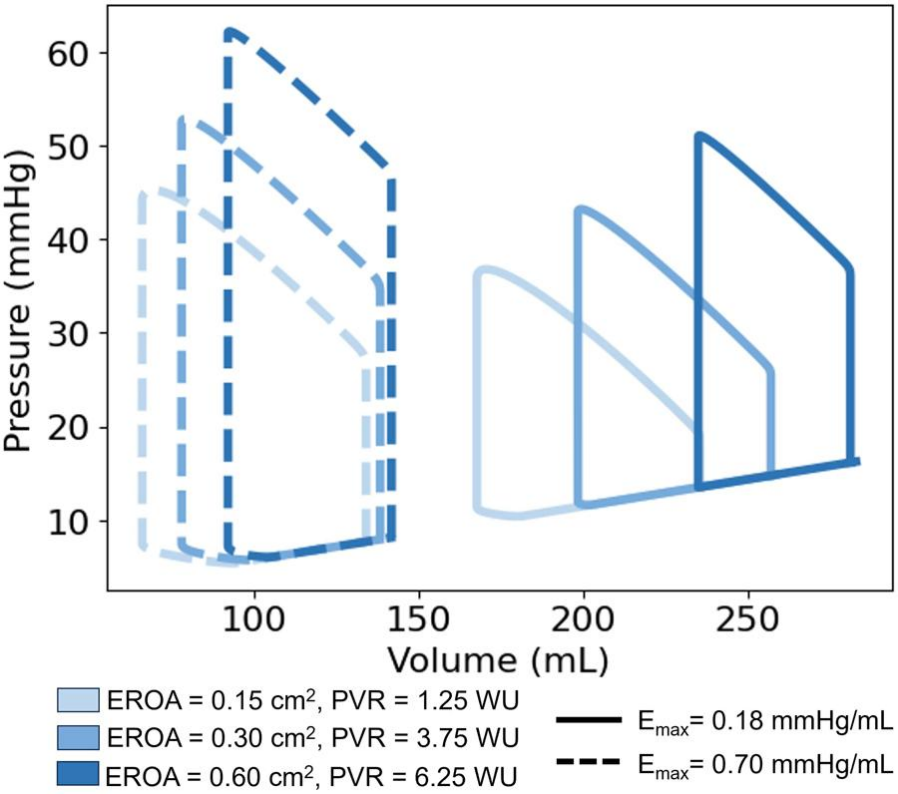
Right ventricle pressure-volume (PV) loops. Comparison of PV Loops with a maximum RV elastance of 0.18 mmHg/mL (solid lines) and 0.7 mmHg/mL (dashed lines). Deepening shades of blue represent larger mitral valve regurgitant orifice areas.

**Figure 8:**
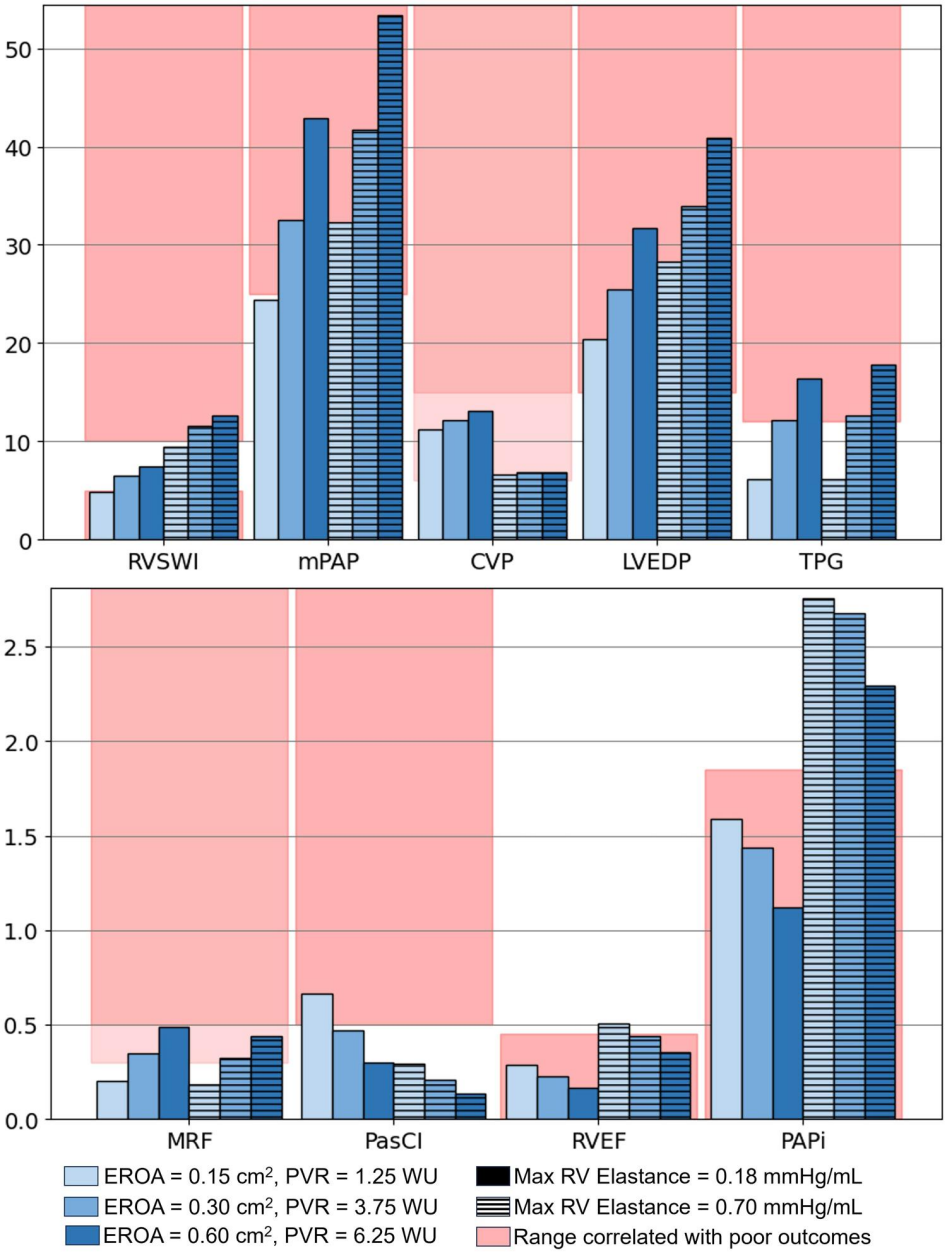
Values of interest to evaluate the risk of varying MR severity on the left heart and RV. Models with increased RV contractility are represented by lined bars and the baseline (lower RV contractility) by solid bars, which is marked with a black box in the legend. RVSWI, right ventricle stroke work index (g/m/beat/$m^{2}$); mPAP, mean pulmonary artery pressure (mmHg); CVP, central venous pressure (mmHg); LVEDP, left ventricle end-diastolic pressure (mmHg); TPG, transpulmonary pressure gradient (mmHg); MRF, mitral regurgitant fraction; PasCI, passive cardiac index (L/min/$m^{2}$); RVEF, right ventricle ejection fraction; PAPi, pulmonary artery pulsatility index.

Clinical metrics associated with RV function and outcomes for varying EROA are presented as the solid bars in Figure 8, where increasing severity is shown with progressively darker blue shades. Increasing EROA decreased RVEF by 24.4% on average. The right ventricle pressure-volume loops for different EROA’s are shown in Figure 7. Greater MR severity increases the right ventricle end diastolic volume and decreases the right ventricle stroke volume, which accounts for the decrease in RVEF. Larger EROAs also increased regurgitant flow into the pulmonary system, raising pulmonary pressures. This led to increases in mPAP, TPG, and other RV-afterload-related metrics shown in Figure 8. In particular, TPG exceeded 12 mmHg for the larger EROAs, and mPAP rose above the 25 mmHg threshold.

### 3.3 RV contractility

To evaluate the mechanistic influence of RV function, maximum RV elastance was increased from 0.18 to 0.7 mmHg/mL to simulate enhanced RV performance. This modification isolated the impact of RV function independent of MV EROA. The corresponding results are shown in Figure 8.

Across all EROA conditions, increasing RV contractility resulted in a 7.0%–9.6% reduction in MRF and a 0.07–0.22 L/min increase in CO. These effects are also evident in Figures 6e, i, where the regurgitant jet at peak systole is reduced relative to baseline. Increasing RV maximum elastance also produced a 6.1% decrease in peak regurgitant jet velocity and an 8.2% decrease in regurgitant volume.

Changes in additional hemodynamic metrics summarized in Table 2 were also observed. Increasing RV elastance led to higher mPAP, as illustrated by the diastolic filling pressures in Figures 6h, l, where the model with elevated RV elastance showed increased filling pressures. Despite similar RV stroke volumes across models, the elevated RV elastance produced a reduction in RVEDV, resulting in an increased RVEF, with values rising to ≥45% across cases.

RV workload increased with higher RV elastance, as reflected by the enlarged PV loop areas in Figure 7a and the corresponding changes in RVSWI shown in Figure 8. For models with mild EROA, RVSWI values fell within a normal or healthy range, whereas in moderate to severe EROA conditions, RVSWI exceeded the upper limit of normal.

Increasing RV contractility also led to an average 44% decrease in CVP, bringing CVP closer to normal ranges. In addition, PAPi substantially increased due to reduced CVP and a smaller difference between sPAP and dPAP. The PasCI decreased with increasing EROA due to reductions in CO but showed substantial reductions with increased RV contractility, reflecting a larger active RV contribution.

Even with elevated RV function, models with worsening EROA showed decreasing RVEF, increasing mPAP, and elevated TPG and PAPi, as shown in Figure 8. Although RV function improved, MRF and regurgitant volume showed only minimal reductions, and EROA remained unchanged.

## 4 Discussion

In this work, we combined image-based 3D flow simulations of the left ventricle, left atrium, and aortic root with a closed-loop 0D cardiovascular model to investigate how mitral regurgitation alters whole-system hemodynamics and right ventricular function. Two patient-specific cases spanning mild and severe ventricular secondary MR were first used to calibrate and validate the framework against clinical data, ensuring that key pressures, flows, and chamber volumes were reproduced. We then used the mild MR case as a baseline to systematically vary mitral valve EROA and RV contractility, to isolate the effects of increasing MR severity and altered RV function on left-heart loading, pulmonary pressures, and RV performance. Across these simulations, we evaluated a panel of clinically relevant metrics, including LVEDP, mPAP, CVP, TPG, RVEF, PAPi, and MR-specific indices, to characterize how MR-related burden and compensatory RV responses jointly shape the risk profile for RV dysfunction. The key findings of this study were threefold. First, progressive mitral regurgitation substantially increased right-ventricular afterload and promoted the development of RV dysfunction, even in cases where intrinsic RV contractile function was augmented. Second, conventional MR severity indices and left-sided cardiac metrics were insufficient to reflect the evolving burden placed on the right heart, as RV impairment could advance despite minimal or stable changes in standard MR descriptors. Finally, these results underscore the importance of directly evaluating RV structure and function in patients with MR, as heightened RV vulnerability appears to be a central determinant of patient risk and has important implications for clinical decision-making and management strategies.

Several computational studies have examined the hemodynamic consequences of mitral regurgitation (MR) using both idealized and patient-specific models. Many have modeled MR in isolated left ventricular or left atrial geometries to characterize regurgitant jet dynamics, leaflet motion, or stroke risk (15, 18). These single-chamber models have been expanded to include coupled LV–LA domains with realistic wall deformation and valve kinematics derived from imaging data, enabling simulation of the temporal evolution of MR under physiological loading conditions and valve repair (13, 18, 41). Computational modeling has also been used to compare different valve prolapse morphologies, degrees of regurgitation, and chamber functional adaptations to MR (13, 15, 42). Furthermore, Zingaro et al. developed a coupled 3D left-heart CFD model with a closed-loop 0D system to simulate cardiac hemodynamics and incorporated MR (14). However, few studies have directly evaluated the impact of MR on the right heart using a framework that is patient-specific not only in the 3D anatomy and motion but also in the 0D representation of the full circulation.

### 4.1 Patient-specific

We modeled two patient-specific cases of mitral regurgitation (mild and severe) with errors across clinically measured parameters ∼10.3%. Patient B had lower systolic pressures (Figures 4c, g) and higher filling pressures (Figures 4d, h) than Patient A. This pattern is often observed as MR severity increases (2). Compared to Patient A, Patient B also exhibited a larger regurgitant jet in the LA (Figures 4a, e) and reverse flow in more pulmonary veins—both of which are characteristic of more severe MR (43). In Patient A, we captured distinct A-wave and E-wave signals, whereas in Patient B, the absence of a recognizable A-wave serves as a marker of severe MR (40). For both patients, representing a range of MR severity, we captured key characteristics of MR in the LH blood flow and in the remaining cardiovascular system.

Mitral regurgitation exists along multiple etiologic pathways, and distinguishing primary from secondary MR is important when interpreting the generalizability of our findings. The patients modeled in this study both exhibited secondary (functional) MR, driven by ventricular remodeling rather than leaflet pathology. Because our simulations prescribe boundary motion from imaging and do not explicitly model leaflet geometry, papillary muscle behavior, or chordae tendineae, the framework is less suited for capturing mechanisms unique to primary MR. Nevertheless, the hemodynamic consequences that drive RV loading (increased regurgitant flow, elevated left atrial pressures, and subsequent pulmonary vascular burden) are shared across primary and secondary MR (44). Thus, while the precise mechanisms differ, the RV vulnerability to increased left-sided pressures identified in this analysis is likely relevant across MR etiologies; however, this will be important to verify with future studies.

Secondary MR can be further classified as atrial or ventricular secondary MR (45, 46). Based on their clinical profiles, both modeled patients are best categorized as having ventricular secondary MR (V-SMR). Although atrial secondary MR (A-SMR) arises from annular dilation due to atrial enlargement rather than ventricular remodeling, both phenotypes result in increased regurgitant volume and elevated left atrial pressure, ultimately transmitting additional load to the pulmonary circulation and RV. While dedicated models of A-SMR may show differences in atrial mechanics or annular dynamics, we anticipate that the RV responses observed here would remain directionally similar, as both A-SMR and V-SMR impose comparable upstream stresses on the right heart. However, future work should extend this framework to A-SMR patients to confirm whether similar RV interactions occur, especially since V-SMR patients are shown to have more severe cardiac dysfunction than A-SMR patients (47).

The clinical status of the modeled patients further informs how MR severity interacts with RV function. Both individuals demonstrated pre-existing pulmonary hypertension and mild tricuspid regurgitation at baseline, indicating that their RVs were already operating under elevated afterload before any simulated changes in MR. Although neither patient had advanced intrinsic RV disease, these comorbidities reduce the RV’s capacity to tolerate additional hemodynamic burden and likely amplify the adverse RV response to worsening MR seen in our simulations.

### 4.2 Mitral regurgitation severity

The MV EROA plays a critical role in determining the severity of MR and the resulting effect on the LH (3). However, it is unclear to what degree MR influences the RV. This work used in-silico modeling to isolate the impact of the MR severity on left heart blood flow and RV function. This was done by modeling three different EROAs (0.18 ${\mathrm{cm}}^{2}$, 0.30 ${\mathrm{cm}}^{2}$, and 0.60 ${\mathrm{cm}}^{2}$) representing cases of mild, moderate, and severe MR.

The increases in regurgitant volume, wider regurgitant jets, and higher E-wave velocities observed with larger EROAs are consistent with increasing severity of mitral regurgitation, where elevated E-wave velocities have been associated with more severe MR (40). The reduction in cardiac output and aortic systolic pressure, together with increases in filling pressures, mirror trends reported in other computational MR studies (15, 48).

Increasing EROA lowered RVEF by an average of 24.4%, reflecting increased RV end-diastolic volume and reduced stroke volume. RVEF values below 45% are considered indicative of RV dysfunction and are strongly correlated with adverse outcomes (5). The modeling results therefore show that greater MR severity compromises RV function.

Larger EROAs also elevated pulmonary pressures, increasing mPAP, TPG, and RV afterload. TPG values above 12 mmHg indicate reactive pulmonary hypertension, which is associated with increased mortality risk (49). In addition, mPAP exceeded the 25 mmHg clinical threshold for pulmonary hypertension. Although RV impairment is only weakly correlated with pulmonary systolic pressure (6), pulmonary hypertension is known to negatively affect RV function (11). When elevated pulmonary pressures coincide with reduced RVEF, the likelihood of RV dysfunction increases substantially (5, 6).

The modeling results, therefore, suggest that increasing EROA, reflecting greater MR severity, simultaneously raises the risk of severe pulmonary hypertension and reduces RV ejection performance. Clinical studies show that the combination of these conditions can lead to right-ventricular dysfunction.

### 4.3 RV contractility

Increasing maximum RV elastance, representing a more functional RV, produced improvements across several RV-associated risk metrics, including CVP, PasCI, RVEF, and PAPi. The decrease in CVP (44% on average) moved values toward a normal range, and PAPi increased into a healthy range due to reduced CVP and a smaller sPAP–dPAP gradient. These changes reflect reductions in systemic venous congestion and increases in RV pulsatility, which are clinically relevant because elevated CVP has been associated with right heart dysfunction and adverse outcomes in LVAD and kidney dysfunction populations (36, 50). Similarly, PAPi values below 1.85 have been linked to RV failure risk (9), and the observed increases in PAPi with enhanced RV function would typically indicate improved RV status.

RV workload also increased with rising RV elastance, as shown by larger PV loop areas and elevated RVSWI values. While RVSWI remained in a normal range for mild EROA, it exceeded healthy thresholds for moderate and severe EROA conditions. Although elevated RVSWI has been associated with worse outcomes in lung transplantation (51), low RVSWI has been linked to RV failure after LVAD implantation and poor outcomes in pulmonary hypertension (52–54), illustrating the mixed clinical interpretation surrounding this metric.

Despite the improved RV function, changes in mitral regurgitation severity were modest. MRF decreased only 7.0%–9.6%, and regurgitant volume decreased by 8.2%, with EROA unchanged. These small improvements occurred even though RV performance increased substantially, with RVEF rising to ≥45%. This mismatch suggests that traditional MR metrics may not strongly reflect RV functional changes. Even with enhanced RV function, worsening MR still led to reductions in RVEF and increases in mPAP, TPG, and PAPi, indicating continued hemodynamic burden on the right heart.

Together, these findings highlight that increased RV contractility improves several RV-specific risk metrics but produces only mild to moderate changes in MR severity. Commonly assessed MR metrics, such as MRF and regurgitant volume, showed limited sensitivity to RV functional improvement, underscoring the need to evaluate RV health independently when assessing MR severity and patient risk. This aligns with recent large-scale clinical studies that have demonstrated that right ventricular dysfunction (RVD) in the setting of severe degenerative MR is a powerful and independent predictor of mortality (55–57).

While several MR metrics—such as EROA, regurgitant volume, and mitral regurgitant fraction—are commonly used to quantify left-sided disease burden, their ability to predict downstream RV dysfunction remains unclear. In our limited cohort, worsening MR severity consistently increased RV afterload, but no single MR descriptor emerged as a reliable predictor of RV decline. Identifying which MR parameters, or combinations thereof, that best correlate with adverse RV responses will likely require analyses across a larger and more heterogeneous patient population. Such studies could help define a subset of patients in whom MR correction is particularly crucial to protect RV function.

### 4.4 Limitations

The study had a few limitations worth noting. Both the aortic and mitral valves were modeled as dynamic orifice planes that regulate flow based on changes in LV volume or pressure-flow driven opening and closing, a simplification consistent with previous studies (16, 58). While incorporating leaflet dynamics would influence flow patterns, particularly by generating additional vortices during filling, we were able to capture key flow characteristics and the impact of mitral regurgitation. Furthermore, this study focused on the broader hemodynamic impacts of mitral regurgitation, which are less sensitive to these localized effects. Furthermore, other studies showed that the level of blood regurgitation is proportional to the EROA and not the type of valve prolapse (15). Given that valve opening and closing occupy less than 5% of the cardiac cycle, mitral valve dynamics were not explicitly modeled; instead, the mitral valve was assumed to open and close instantaneously (13, 59).

Furthermore, the analysis was performed using only two patient-specific models. Future studies incorporating a broader range of patients, particularly those with atrial secondary MR or with normal pulmonary pressures, will be necessary to account for population heterogeneity and validate the robustness of these findings.

Another limitation of our model was the use of prescribed boundary motion for the left ventricle, left atrium, and aorta. This approach prevents changes in ventricular and atrial volumes or dynamics in response to varying MR severity or right ventricular (RV) function. This may lead to an overestimation of LVEDP or pulmonary pressures with increasing MR severity. Furthermore, prescribing boundary motion limited the model’s ability to capture adaptive structural changes in response to disease progression, such as dilation of the LA and LV with greater MR. Nonetheless, this constraint allowed us to systematically isolate the effects of EROA, RV contractility, and heart rate on cardiovascular hemodynamics without confounding influences from structural remodeling or compensatory mechanisms.

Finally, the cardiovascular system is a complex, adaptive network that responds to physiological changes. Our models did not account for all these adaptive responses, indicating the need for further research to fully characterize these dynamics and appropriately integrate them into the model. In the event of worsening mitral regurgitation (MR), the heart undergoes both acute and chronic adaptations. Acutely, MR can lead to sudden volume overload, causing increases in left atrial and pulmonary pressures, potentially resulting in pulmonary edema and acute heart failure. While we increased pulmonary vascular resistance (PVR) with worsening MR based on trends reported in clinical studies (32, 33), the magnitude of PVR change for any individual patient is unknown. As a result, our models may overestimate or underestimate the true RV afterload. Nonetheless, the applied adjustments fall within physiologically reasonable ranges and capture an important component of the adaptive response commonly observed in patients. Increased pulmonary artery pressure also leads to stretching of the pulmonary artery’s elastic fibers, resulting in reduced compliance, though quantifying this reduction is challenging due to individual variability and the complexity of involved factors (33, 60). For example, although the 0D model is calibrated to patient-specific data, it does not incorporate the adaptive adjustments that occur as the RV–PA system remodels over time. In a physiological setting, increases in RV contractility (Ees) are often accompanied by changes in arterial elastance (Ea) to maintain ventricular–vascular matching, but our model held pulmonary vascular properties fixed for each MR severity. As a result, variations in Ees did not elicit compensatory changes in Ea, and some simulated conditions—particularly at low contractility—may represent partially uncoupled states that would differ from an adaptive system. While prior work has begun to explore rules governing RV–PA adaptation, how these mechanisms evolve across the spectrum of MR remains incompletely understood, and integrating such adaptive behavior is an important direction for future work. Persistent volume overload from MR induces LV dilation and eccentric hypertrophy as compensatory mechanisms to maintain forward stroke volume. Over time, these adaptations may become maladaptive, leading to decreased contractility and progression to heart failure. Additionally, the right ventricle (RV) may experience increased afterload due to elevated pulmonary pressures, resulting in RV remodeling and dysfunction (61).

Failing to incorporate these adaptive mechanisms into computational models may limit their accuracy in predicting cardiovascular responses under various conditions. Therefore, further research is essential to integrate these complex dynamics into models for a better understanding and prediction of cardiovascular behavior.

## 5 Conclusion

In this study, we developed a patient-specific computational model to analyze mitral regurgitation and its systemic effects. Dynamic contrast-enhanced CT images were used to prescribe boundary motion, and a 3D model of the left ventricle, left atrium, and aortic root was coupled with a closed-loop lumped parameter model representing the remainder of the cardiovascular system. Parameters of the 0D model were optimized using patient-specific clinical data to accurately replicate global hemodynamics.

We then systematically modified the patient-specific model to isolate and evaluate the effects of changes in mitral regurgitation severity and right ventricular contractility. These variations allowed us to explore the individual and combined effects of MR-related burden and compensatory mechanisms on RV function.

Our findings indicate that worsening mitral regurgitation significantly threatens RV health, even when RV contractility improves, highlighting the vulnerability of the RV to elevated left-sided pressures and volume overload. Furthermore, left heart–centric metrics or MR-specific severity indices alone do not adequately reflect RV condition, underscoring the importance of independently assessing RV function when evaluating the clinical impact and risk profile of MR. These findings support the consideration of whole-heart assessment approaches in future research and clinical evaluation of mitral regurgitation.

## Data Availability

The raw data supporting conclusions for this study will be available on reasonable request from the corresponding author.
