# Supplementary material for: Image-based, whole-system hemodynamic modeling of mitral regurgitation and its impact on the right ventricular function
Source: Front Cardiovasc Med. 2026 Jan 30;12:1673443. doi: 10.3389/fcvm.2025.1673443 (PMC12902947; doi:10.3389/fcvm.2025.1673443)
Supplement: Supplementary file 1 [file Datasheet1.pdf]

Supplementary Material

1 ZERO-DIMENSIONAL MODEL

The 0D model features the major components of the cardiovascular system. The valves are modeled as bi-resistive elements that are functions of the pressure difference across the valve. The pressure-volume relationship for the right atrium and right ventricle was molded with:

$$p(t) = E(t) \cdot V(t), \tag{S1}$$

where p is the pressure in the chamber, V is the volume of the chamber, and the time-varying elastance element is:

$$E(y) = (E_{max} - E_{min}) \cdot y(t) + E_{min}, \tag{S2}$$

where $y(t)$ is the time-dependant activation function for the chamber and E_{max} and E_{min} denote the maximum and minimum chamber elastance, respectively. The pressure-flow relationship throughout the whole cardiovascular system is described using first-order ordinary differential equations (ODE). These governing equations of the 0D cardiovascular system are described with the following equations:

left heart and systemic circulation

$-Q^{\ell}_{\mathrm{at}} - \sum_{i=1}^{n^{\mathrm{pul}}_{\mathrm{ven}}} q^{\mathrm{pul}}_{\mathrm{ven},i} + q^{\ell}_{\mathrm{v,in}} = 0$	left atrium flow balance
$\frac{1}{\tilde{R}^{\ell}_{\mathrm{v,in}}}(p^{\ell}_{\mathrm{at}} - p^{\ell}_{\mathrm{v}}) - q^{\ell}_{\mathrm{v,in}} = 0$	mitral valve momentum
$-Q^{\ell}_{\mathrm{v}} - q^{\ell}_{\mathrm{v,in}} + q^{\ell}_{\mathrm{v,out}} = 0$	left ventricle flow balance
$\frac{1}{\tilde{R}^{\ell}_{\mathrm{v,out}}}(p^{\ell}_{\mathrm{v}} - p^{\mathrm{sys}}_{\mathrm{ar}}) - q^{\ell}_{\mathrm{v,out}} = 0$	aortic valve momentum
$-Q^{\mathrm{sys}}_{\mathrm{aort}} - q^{\ell}_{\mathrm{v,out}} + q^{\mathrm{sys}}_{\mathrm{ar,p}} = 0$	aortic root flow balance
$Z^{\mathrm{sys}}_{\mathrm{ar}}\, q^{\mathrm{sys}}_{\mathrm{ar,p}} - p^{\mathrm{sys}}_{\mathrm{ar}} + p^{\mathrm{sys}}_{\mathrm{ar,d}} = 0$	aortic root inertia
$C^{\mathrm{sys}}_{\mathrm{ar}} \frac{\mathrm{d}p^{\mathrm{sys}}_{\mathrm{ar,d}}}{\mathrm{d}t} - q^{\mathrm{sys}}_{\mathrm{ar,p}} + q^{\mathrm{sys}}_{\mathrm{ar}} = 0$	systemic arterial flow balance
$L^{\mathrm{sys}}_{\mathrm{ar}} \frac{\mathrm{d}q^{\mathrm{sys}}_{\mathrm{ar}}}{\mathrm{d}t} + R^{\mathrm{sys}}_{\mathrm{ar}}\, q^{\mathrm{sys}}_{\mathrm{ar}} - p^{\mathrm{sys}}_{\mathrm{ar,d}} + p^{\mathrm{sys}}_{\mathrm{ven}} = 0$	systemic arterial momentum
$C^{\mathrm{sys}}_{\mathrm{ven}} \frac{\mathrm{d}p^{\mathrm{sys}}_{\mathrm{ven}}}{\mathrm{d}t} - q^{\mathrm{sys}}_{\mathrm{ar}} + q^{\mathrm{sys}}_{\mathrm{ven}} = 0$	systemic venous flow balance
$R^{\mathrm{sys}}_{\mathrm{ven}}\, q^{\mathrm{sys}}_{\mathrm{ven}} - p^{\mathrm{sys}}_{\mathrm{ven}} + p^{r}_{\mathrm{at}} = 0$	systemic venous momentum

right heart and pulmonary circulation

$$\frac{\mathrm{d}}{\mathrm{d}t}(\frac{p_{\mathrm{at}}^{r}}{E_{\mathrm{at}}^{r}}) - q_{\mathrm{ven}}^{\mathrm{pul}} + q_{\mathrm{v,in}}^{r} = 0 \qquad \text{right atrium flow balance}$$

$$\frac{1}{\tilde{R}_{\mathrm{v,in}}^{r}}(p_{\mathrm{at}}^{r} - p_{\mathrm{v}}^{r}) - q_{\mathrm{v,in}}^{r} = 0 \qquad \text{tricuspid valve momentum}$$

$$\frac{\mathrm{d}}{\mathrm{d}t}(\frac{p_{\mathrm{v}}^{r}}{E_{\mathrm{v}}^{r}}) - q_{\mathrm{v,in}}^{r} + q_{\mathrm{v,out}}^{r} = 0 \qquad \text{right ventricle flow balance}$$

$$\frac{1}{\tilde{R}_{\mathrm{v,out}}^{r}}(p_{\mathrm{v}}^{r} - p_{\mathrm{ar}}^{\mathrm{pul}}) - q_{\mathrm{v,out}}^{r} = 0 \qquad \text{pulmonary valve momentum}$$

$$C_{\mathrm{ar}}^{\mathrm{pul}}\frac{\mathrm{d}p_{\mathrm{ar}}^{\mathrm{pul}}}{\mathrm{d}t} - q_{\mathrm{v,out}}^{r} + q_{\mathrm{ar}}^{\mathrm{pul}} = 0 \qquad \text{pulmonary arterial flow balance}$$

$$R_{\mathrm{ar}}^{\mathrm{pul}}\, q_{\mathrm{ar}}^{\mathrm{pul}} - p_{\mathrm{ar}}^{\mathrm{pul}} + p_{\mathrm{ven}}^{\mathrm{pul}} = 0 \qquad \text{pulmonary arterial momentum}$$

$$C_{\mathrm{ven}}^{\mathrm{pul}}\frac{\mathrm{d}p_{\mathrm{ven}}^{\mathrm{pul}}}{\mathrm{d}t} - q_{\mathrm{ar}}^{\mathrm{pul}} + \sum_{i=1}^{n_{\mathrm{ven}}^{\mathrm{pul}}} q_{\mathrm{ven},i}^{\mathrm{pul}} = 0 \qquad \text{pulmonary venous flow balance}$$

$$R_{\mathrm{ven},i}^{\mathrm{pul}}\, q_{\mathrm{ven},i}^{\mathrm{pul}} - p_{\mathrm{ven}}^{\mathrm{pul}} + p_{\mathrm{at},i}^{\ell} = 0 \qquad \text{pulmonary venous momentum}$$

where,

$$Q_{\mathrm{at}}^{\ell} := -\frac{\mathrm{d}V_{\mathrm{at}}^{\ell}}{\mathrm{d}t}, \quad Q_{\mathrm{v}}^{\ell} := -\frac{\mathrm{d}V_{\mathrm{v}}^{\ell}}{\mathrm{d}t}, \tag{S3}$$

For the 3D-0D model coupling, the flux (Q) from the 3D model becomes a primary variable of the ODE system and is enforced with a Lagrange multiplier constraint (1).

The flow-pressure relations for healthy valves in the 0D model are defined by the pressure difference across the valve,

$$q(p - p_{\mathrm{open}}) = \frac{p - p_{\mathrm{open}}}{\tilde{R}}, \quad \text{with } \tilde{R} = \begin{cases} R_{\mathrm{max}}, & p < p_{\mathrm{open}} \\ R_{\mathrm{min}}, & p \geq p_{\mathrm{open}} \end{cases}$$

where p is the pressure upstream of the valve and p_{open} is the pressure downstream of the valve. For modeling valve regurgitation in the 0D model, the following from Franz et al. is used,

$$q(p - p_{\mathrm{open}}) = \begin{cases} cA_{\mathrm{o}}\sqrt{p - p_{\mathrm{open}}}, & p < p_{\mathrm{open}} \\ \frac{p - p_{\mathrm{open}}}{R_{\mathrm{min}}}, & p \geq p_{\mathrm{open}} \end{cases}$$

where R_{max} =1e^{7} Pa·s/mm^{3}, R_{min} =1e^{-5} Pa·s/mm^{3}, c=37.2 $\frac{mm/s}{sqrt(Pa)}$, and A_o is the regurgitant orifice area (2).

The following values were fit for Patient A and Patient B's 0D parameters (Table S1).

0D Parameter	Pat. A	Pat. B
LA Minimum Elastance, $E_{\min}^{\mathrm{LA}}$ (Pa/mm^3)	1.80e-2	1.99e-2
LA Maximum Elastance, $E_{\max}^{\mathrm{LA}}$ (Pa/mm^3)	2.25e-2	2.01e-2
MV Regurgitant Orifice Area, MV_{ROA} (cm^2)	0.18	0.49
LV Minimum Elastance, $E_{\min}^{\mathrm{LV}}$ (Pa/mm^3)	1.11e-2	1.43e-2
LV Maximum Elastance, $E_{\max}^{\mathrm{LV}}$ (Pa/mm^3)	10.2e-2	4.91e-2
Systemic Artery Impedance, $Z_{\mathrm{ar}}^{\mathrm{sys}}$ (Pa·s/mm^3)	0.99e-02	1.34e-2
Systemic Artery Capacitance, $C_{\mathrm{ar}}^{\mathrm{sys}}$ (mm^3/Pa·s)	98.8	17.5
Systemic Artery Resistance, $R_{\mathrm{ar}}^{\mathrm{sys}}$ (Pa·s/mm^3)	8.63e-02	9.16e-2
Systemic Vein Capacitance, $C_{\mathrm{ven}}^{\mathrm{sys}}$ (mm^3/Pa·s)	752.6	381.0
Systemic Vein Resistance, $R_{\mathrm{ven}}^{\mathrm{sys}}$ (Pa·s/mm^3)	6.24e-02	4.64e-2
RA Minimum Elastance, $E_{\min}^{\mathrm{RA}}$ (Pa/mm^3)	0.90e-2	0.95e-2
RA Maximum Elastance, $E_{\max}^{\mathrm{RA}}$ (Pa/mm^3)	1.40e-2	1.39e-2
RV Minimum Elastance, $E_{\min}^{\mathrm{RV}}$ (Pa/mm^3)	0.77e-02	1.03e-2
RV Maximum Elastance, $E_{\max}^{\mathrm{RV}}$ (Pa/mm^3)	2.90e-02	3.36e-2
Pulmonary Artery Capacitance, $C_{\mathrm{ar}}^{\mathrm{pul}}$ (mm^3/Pa·s)	21.0	5.75
Pulmonary Artery Resistance, $R_{\mathrm{ar}}^{\mathrm{pul}}$ (Pa·s/mm^3)	1.38e-02	0.804e-2
Pulmonary Vein Capacitance, $C_{\mathrm{ven}}^{\mathrm{pul}}$ (mm^3/Pa·s)	537.5	160.9
Pulmonary Vein Resistance, $R_{\mathrm{ven}}^{\mathrm{pul}}$ (Pa·s/mm^3)	0.10e-03	0.83e-3

Table S1. The 0D model parameters fit during optimization for Patient A and Patient B

2 RESULTS AND DISCUSSION

2.1 Chronotropic Response

An increased heart rate (HR) is a common compensatory mechanism for maintaining CO with MR (3). To study the impact of a chronotropic response, we modulated the heart rate to achieve a similar CO as the baseline model (3.75 L/min). This was optimized using the 0D model (Fig. 2a). The duration of systole and diastole was adjusted as described in Chung et al. (4). This was done for two models with a maximum RV elastance of 0.18 mmHg/mL and EROAs of 0.30 cm^2 (moderate) and 0.60 cm^2 (severe). To achieve proper dynamics of the LV, LA, and aorta, the boundary velocity was scaled by the ratio of the baseline model's timestep and the new timestep ($\mathrm{dt_{baseline}}/\mathrm{dt_{new}}$). The results and discussion for this analysis can be found in the Supplementary Material.

The patient-specific case of mild MR had a cardiac output of 3.75 mL/min. The larger EROA was associated with a reduced cardiac output of 3.06 L/min and 2.41 L/min for the moderate and severe cases, respectively. However, under physiological conditions, the cardiovascular system adapts to maintain CO through various mechanisms, such as a chronotropic response, where an increase in heart rate (HR) can help to preserve CO (3). This adaptive mechanism is reflected in the comparison of patients A and B, who had mild MR and an HR of 53 bpm and severe MR and an HR of 120 bpm, respectively.

The chronotropic response was incorporated by increasing the HR in the models with moderate and severe EROA. For the moderate MR case, the HR was increased to 69 bpm, increasing the CO from 3.06 to 3.87 L/min. For the severe MR model, the HR was adjusted to 79 bpm, yielding a CO of 3.57 L/min. These

fall within 5% of the baseline models CO (3.75 L/min). The peak regurgitant velocity for the moderate case was 4.48 m/s and 4.57 m/s for the severe case. Due to the increase in heart rate, the severe MR model exhibits a slightly higher regurgitant peak velocity than the moderate MR model, despite having a larger EROA.

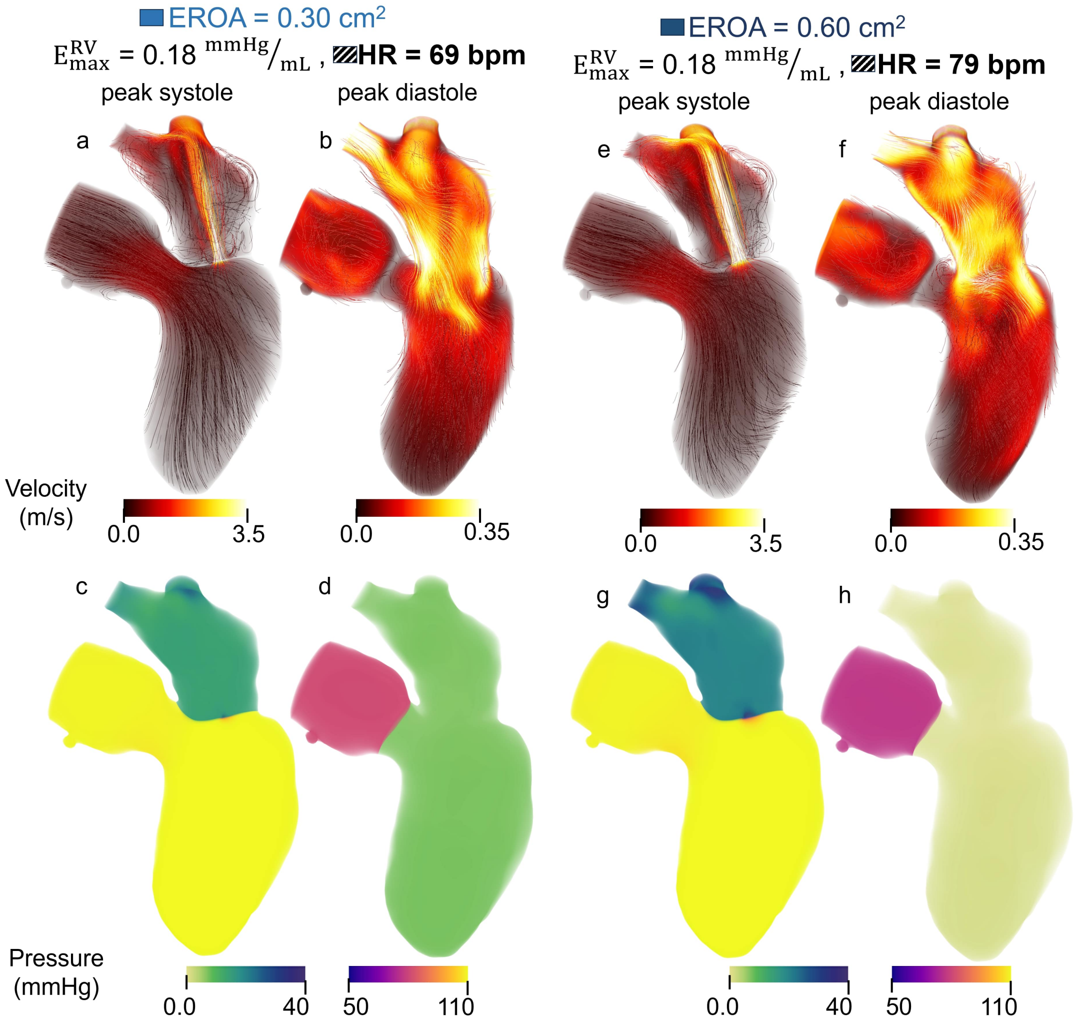

Figure S1. Velocity and Pressure at peak systole and diastole for models with an EROA of 0.30 cm^2 and HR of 69 bpm (left) and an EROA of 0.60 cm^2 and HR of 79 bpm (right).

Figure S3 presents the results of these models with and without the chronotropic response. As expected, the increased HR significantly augmented the CO, leading to a corresponding rise in systemic pressures by 14.0% and 32.4%, for the moderate and severe MR models, respectively. Since the CO was preserved, the PasCI exhibited a less pronounced reduction than models without HR adaptation. This suggests that with a chronotropic response, the EROA has a significantly reduced impact on the degree of passive RV function. In turn, the contractility of the RV plays a much larger role (Figure 7).

Compared to the baseline model, models with preserved CO and worsening mitral regurgitation, showed an increased mPAP, LVEDP, and mitral regurgitation fraction, which aligns with clinical expectations. And while the drop in RVEF and increase in mPAP was less pronounced than in the cases without a chronotropic response, results still indicate risk to the RV.

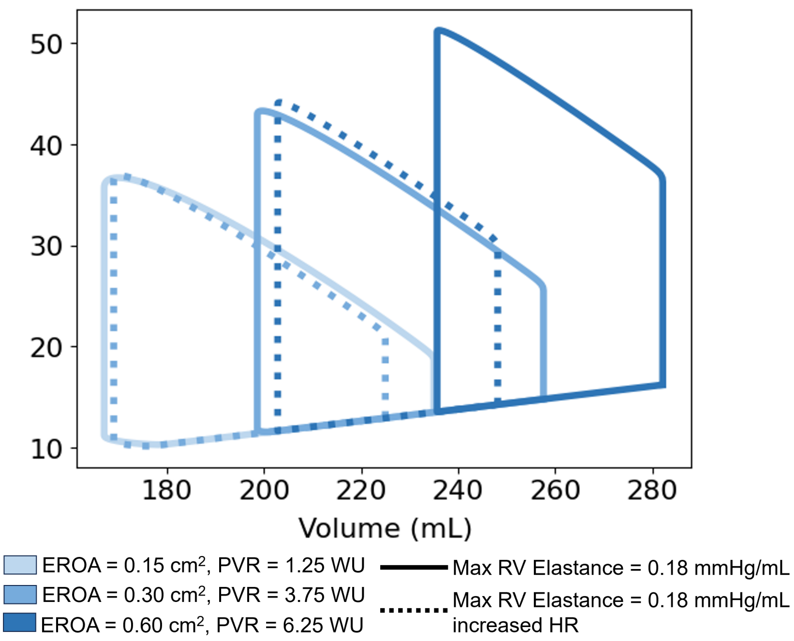

Figure S2. Right Ventricle Pressure-Volume (PV) Loops. Comparison of PV loops with no chronotropic response (solid lines) and with a chronotropic response (increased heart rate, dotted lines). Deepening shades of blue represent larger mitral valve regurgitant orifice areas.

An elevated HR reduces the duration of diastole while maintaining the length of systole (4). Consequently, there is less time for right ventricular (RV) diastolic filling, which decreases RV pressures, as well as RV end-diastolic volume (RVEDV) and RV end-systolic volume (RVESV) (Fig. S2). However, this effect was not observed in the left heart (LH) due to the modeling approach. The 3D left heart model was formulated as a boundary-driven Navier-Stokes problem with an ALE framework. Therefore, while we increased the heart rate, the volumes of the left ventricle and atrium remained unchanged. It is important to note that our model did not include the physiological adaptations typically seen with acute HR increases, such as those occurring during exercise. These adaptations, such as increased vascular resistance, decreased vessel compliance, and enhanced myocardial contraction, were not incorporated (5, 6, 7). As a result, our simulations did not produce increases in mean pulmonary artery pressure or left ventricular end-diastolic pressure that often accompany acute HR elevations.

REFERENCES

1. Hirschvogel M, Bonini M, Balmus M, Nordsletten D. Effective block preconditioners for fluid dynamics coupled to reduced models of a non-local nature. *Computer Methods in Applied Mechanics and Engineering* **435** (2025) 117541. doi:10.1016/J.CMA.2024.117541.

2. Franz J, Czechowicz K, Waechter-Stehle I, Hellmeier F, Razafindrazaka F, Kelm M, et al. An orifice shape-based reduced order model of patient-specific mitral valve regurgitation. *Engineering Applications of Computational Fluid Mechanics* **15** (2021) 1868–1884. doi:10.1080/19942060.2021.1995048/SUPPL_FILE/TCFM_A_1995048_SM3442.ZIP.

3. Dimopoulos K, Alonso-Gonzalez R, D'Alto M. 7 - heart failure, exercise intolerance, and physical training. Gatzoulis MA, Webb GD, Daubeney PE, editors, *Diagnosis and Management of Adult Congenital Heart Disease (Third Edition)* (Elsevier). Third edition edn. (2018), 77–87. doi:https://doi.org/10.1016/B978-0-7020-6929-1.00007-1.

4. Chung CS, Karamanoglu M, Kovács SJ. Duration of diastole and its phases as a function of heart rate during supine bicycle exercise. *American Journal of Physiology - Heart and Circulatory Physiology* **287** (2004) 2003–2008. doi:10.1152/AJPHEART.00404.2004/ASSET/IMAGES/LARGE/ZH40100433900004.JPEG.

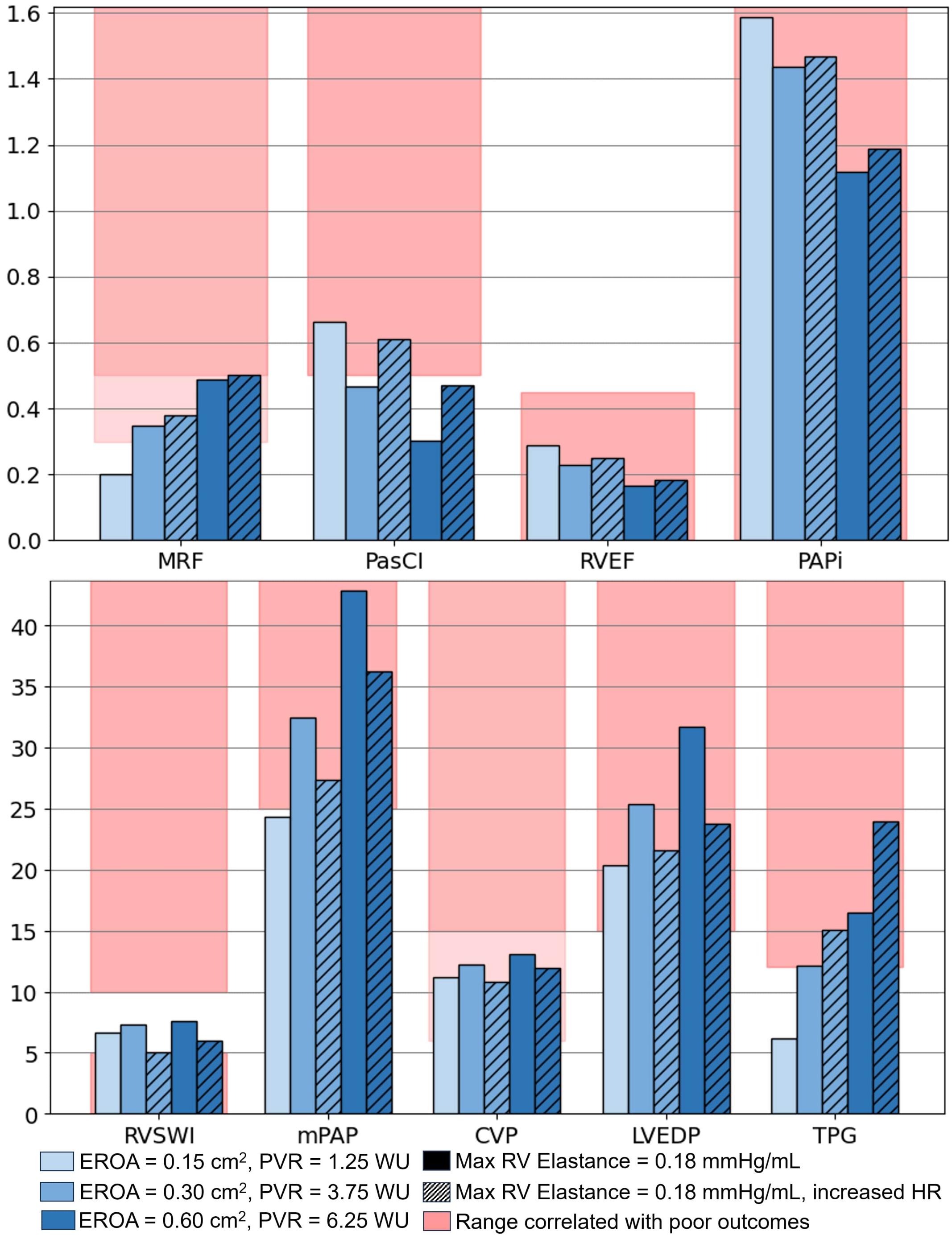

Figure S3. Values of interest to evaluate the risk of increased MR severity with a chronotropic response on the LH and RV. Solid bars are without a chronotropic response and diagonally dashed bars are with a chronotropic response. The lightest blue, solid bar reports the results of the baseline model.

5. Schnell F, Claessen G, Gerche AL, Claus P, Bogaert J, Delcroix M, et al. Atrial volume and function during exercise in health and disease. *Journal of Cardiovascular Magnetic Resonance* **19** (2016) 104. doi:10.1186/S12968-017-0416-9.

6. Yuchi Y, Suzuki R, Kanno H, Saito T, Teshima T, Matsumoto H, et al. Influence of heart rate on right ventricular function assessed by right heart catheterization and echocardiography in healthy anesthetized dogs. *BMC Veterinary Research* **18** (2022) 166. doi:10.1186/S12917-022-03271-Y.

7. Wright SP, Opotowsky AR, Buchan TA, Esfandiari S, Granton JT, Goodman JM, et al. Flow-related right ventricular to pulmonary arterial pressure gradients during exercise. *Cardiovascular Research* **115** (2019) 222–229. doi:10.1093/CVR/CVY138.
